# IoT-blockchain empowered Trinet: optimized fall detection system for elderly safety

**DOI:** 10.3389/fbioe.2023.1257676

**Published:** 2023-09-21

**Authors:** Fayez Alfayez, Surbhi Bhatia Khan

**Affiliations:** ^1^ Department of Computer Science and Information, College of Science, Majmaah University, Al-Majmaah, Saudi Arabia; ^2^ Department of Data Science, School of Science, Engineering and Environment, University of Salford, Salford, United Kingdom

**Keywords:** IoT, blockchain, elderly people, deep learning, SSA, TLBO

## Abstract

Numerous elderly folks reside alone in their homes. Seniors may find it difficult to ask for assistance if they fall. As the elderly population keeps growing, elderly fall incidents are becoming a critical public health concern. Creating a fall detection system for the elderly using IoT and blockchain is the aim of this study. Data collection, pre-processing, feature extraction, feature selection, fall detection, and emergency response and assistance are the six fundamental aspects of the proposed model. The sensor data is collected from wearable devices using elderly such as accelerometers and gyroscopes. The collected data is pre-processed using missing value removal, null value handling. The features are extracted after pre-processed data using statistical features, autocorrelation, and Principal Component Analysis The proposed approach utilizes a novel hybrid HSSTL combines Teaching-Learning-Based Optimization and Spring Search Algorithm to select the optimal features. The proposed approach employs TriNet, including Long Short-Term Memory, optimized Convolutional Neural Network (CNN), and Recurrent Neural Network for accurate fall detection. To enhance fall detection accuracy, use the optimized Convolutional Neural Network obtained through the hybrid optimization model HSSTL. Securely store fall detection information in the Blockchain network when a fall occurs. Alert neighbours, family members, or those providing immediate assistance about the fall occurrence using Blockchain network. The proposed model is implemented in Python. The effectiveness of the suggested model is evaluated using metrics for accuracy, precision, recall, sensitivity, specificity, f-measure, NPV, FPR, FNR, and MCC. The proposed model outperformed with the maximum accuracy of 0.974015 at an 80% learning rate, whereas the suggested model had the best accuracy score of 0.955679 at a 70% learning rate.

## 1 Introduction

The world’s expanding elderly population is known to be one of the main causes of physical, psychological, and financial problems, and an exponential rise in falls is widely acknowledged as one of these problems (Shahzad and Kim, 2018). The most effective technique to lessen the severe effects of a fall while ensuring user convenience is through an IoT-based wearable solution (Gia et al., 2018). Several wearable fall detection systems have been developed on the basis of machine learning models to provide emergency alarms and services to improve safety and health-related quality of life (Liu et al., 2018). Context-aware systems and wearable technology make up the two kinds of fall detection (FD) systems. FD has been extensively studied using context-aware systems (video systems). Wearable technology must be adopted, though, given the large number of elderly people and their desire to live freely in their own homes (Khojasteh et al., 2018). The most common direct effects of falls are fractures and other chronic ailments, which can result in disability and loss of freedom, as well as psychological fear of falling again (Clemente et al., 2019). Elderly adults who fall suffer from slight to serious injuries in addition to financial pressure and mental stress (Chen et al., 2020). Falls are the most common accident cause for senior citizens who live independently. Timely and accurate fall detection is crucial to reducing injuries and preventing fatalities (Lee and Tseng, 2019). Fall Detection Systems (FDSs) have as their primary goal the implementation of online (continuous) monitoring of vulnerable persons to detect happening falls and thereafter issue automatic help requests so that timely assistance may be provided (Musci et al., 2018). A significant public health danger for senior people around the world is falling.

The system automatically responds by sending messages to the organisations in charge of caring for the elderly when a fall is detected (Yacchirema et al., 2018). According to US Centres for the World Health Organisation (“WHO”), falls are the second greatest cause of unintentional injury mortality, with an older patient being admitted to the hospital every 11 s. Wearable devices, ambient devices, and vision-based sensors are the three basic categories into which automatic fall detection systems fall (Zitouni et al., 2019). When an accident like a fall happens, fall detection aims to automate the alarms and notification system and offer aid. Automatic fall detection and alerting the designated carer are the main goals of fall detection apps (Al-Rakhami et al., 2021). Over the past 20 years, a number of fall detection techniques have been investigated and discovered. For fall detection and prevention, different algorithms and sensor types (such as wearable, environmental, and visual) have been examined (Al Nahian et al., 2021). The urgency of fall detection for senior safety is underscored by alarming statistics revealing its substantial impact on public health. Research indicates that falls are a leading cause of injury-related hospitalizations and fatalities among the elderly population. Each year, a significant percentage of senior’s experience fall incidents, imposing not only physical harm but also burdening healthcare resources. In light of these compelling facts, our research endeavors to develop a robust fall detection system. The imperative for fall detection systems among seniors gains further weight when considering the sobering statistical landscape and its consequential impact on healthcare resources. Statistics reveal that falls constitute a significant public health challenge, with one out of every four older adults experiencing a fall each year. These incidents lead to more than 3 million emergency department visits, resulting in over 800,000 hospitalizations annually in the United States alone.

Elderly healthcare issues have received a lot of attention, particularly fall accidents because they can result in fractures and have catastrophic repercussions. Therefore, it is crucial for both older persons and those who care for them to effectively detect fall accidents (Lu and Chu, 2018). A collection of sophisticated fall detection systems for the elderly must be created. Gyroscopes and accelerometers, which are inertial sensors, are used to monitor old people’s movement. Some researchers incorporate the sensors in shoes to make it easier to wear the sensor and prevent it from interfering with daily living. Digital cameras are typically employed to record falls in order to increase the detection accuracy (Liu et al., 2020). In order to employ classifiers or other types of AI to determine the patient’s present condition, wearable devices are focused on the user wearing some sort of gadget with embedded sensors that monitor changes in posture and body movement (Taramasco et al., 2018). The rise in elderly living alone highlights the urgent public health issue of fall incidents among them. Such falls have dire consequences, leading to hospitalizations and fatalities. Existing fall detection systems often suffer from delays, false alarms, and inadequate notifications. To address these shortcomings, this study proposes an innovative solution combining IoT, deep learning, and blockchain for accurate and timely fall detection, significantly enhancing elderly safety.

The motivation behind developing the IoT-Blockchain Empowered TriNet, an optimized fall detection system for elderly safety, is driven by the pressing need to ensure the wellbeing and security of older adults in an aging society. Falls among the elderly population pose significant risks, including severe injuries, reduced quality of life, and increased healthcare costs. As the population continues to age, it becomes imperative to develop innovative solutions that empower older adults to lead independent lives while providing peace of mind to their families and caregivers. The choice of the IoT-Blockchain Empowered TriNet system stems from its unique features and capabilities, which make it a promising solution in addressing the safety concerns of the elderly: Firstly, the system integrates advanced sensors within the living environment of older adults, utilizing the power of IoT technology. These sensors are capable of detecting changes in motion, orientation, and environmental conditions, enabling precise and real-time fall detection. Secondly, the TriNet system continuously monitors the data collected by the sensors, leveraging intelligent algorithms to analyze patterns and identify potential falls. In the event of a fall, immediate alerts are sent to caregivers, family members, and emergency services, facilitating prompt assistance and reducing response times. Thirdly, the system’s combination of IoT technology with blockchain enhances accuracy and reliability. The distributed ledger of blockchain ensures secure and tamper-proof data storage, increasing trust among stakeholders and enhancing the overall reliability of fall detection. Moreover, the TriNet system prioritizes privacy by design, safeguarding sensitive health information through secure storage and authorized access. The decentralized nature of blockchain technology further strengthens security measures, reducing the risk of data breaches and unauthorized access. Additionally, the vast amount of data collected by the TriNet system can be utilized for long-term analysis, providing valuable insights into fall patterns, risk factors, and preventive measures. This data-driven approach empowers healthcare professionals and researchers to develop tailored interventions and improve the overall wellbeing of the elderly population.

This study has three primary objectives. Firstly, it aims to create a robust fall detection system utilizing IoT sensor data and advanced machine learning techniques, such as Long Short-Term Memory (LSTM), optimized Convolutional Neural Network (CNN), and Recurrent Neural Network (RNN). The system’s purpose is to swiftly identify fall incidents, enhancing the safety of elderly individuals. Secondly, the research strives to establish a secure and efficient blockchain-based mechanism. This mechanism not only securely stores fall detection data but also enables prompt response and aid in case of falls. By leveraging this blockchain network, emergency care providers, neighbors, and family members can be quickly notified, allowing for timely intervention. Thirdly, the performance of the proposed model will be comprehensively validated using a diverse dataset and an array of evaluation metrics including accuracy, precision, recall, sensitivity, specificity, F-measure, Negative Predictive Value (NPV), False Positive Rate (FPR), False Negative Rate (FNR), and Matthews Correlation Coefficient (MCC).

Additionally, this study pursues several secondary objectives. One focus is on investigating the efficacy of various feature extraction methods, encompassing statistical features, autocorrelation, and Principal Component Analysis (PCA). These methods are explored to effectively capture crucial information from wearable sensor data. Furthermore, the research delves into a hybrid optimization approach, merging the Spring Search Algorithm (SSA) and Teaching-Learning-Based Optimization (TLBO), to conduct feature selection. The impact of this approach on model performance is compared with traditional feature selection methods. An in-depth analysis of the individual contributions of components within the TriNet model, which includes LSTM, optimized CNN, and RNN, will offer insights into their distinct roles in bolstering fall detection accuracy. Lastly, the study assesses the practical feasibility of implementing the proposed system in real-world scenarios. This assessment factors in aspects such as device compatibility, battery durability, and the mitigation of false positives and negatives. Through these objectives, the study strives to address the critical concern of fall incidents among the elderly using innovative technology solutions.

The foremost contribution of the research work is:

• To identify the optimal features from the extracted set, the feature selection phase utilizing a hybrid optimization model HSSTL. The goal is to select the most relevant features for the fall detection system using this hybrid model.
• The integration of TriNet, which includes the LSTM, optimised CNN, and RNN models, is suggested as a means of achieving accurate fall detection. In order to capture temporal dependencies, the LSTM model is used, and in order to extract spatial data, the optimised CNN is used. Sequence modelling also makes use of the RNN model.
• To enhance fall detection accuracy, the optimized CNN is obtained through the hybrid optimization model HSSTL.

The remaining sections of this essay are structured as follows: The literature on fall detection systems for elderly safety is included in Section 2. Section 3 goes over the suggested approach. Additionally, Section 4 describes the results obtained using the projected model, and Section 5 wraps up this research.

## 2 Literature review

Falls have drawn a lot of interest from the scholarly and business areas. Accuracy of technological fall detection methods varies. In an effort to reduce falls, monitoring everyday activities without body-attached sensors has been investigated. Accelerometry-based methods concentrate on identifying falls through modifications in body alignment following a significant negative acceleration. There is not, however, a fall detection system that is 100 percent accurate and free of false alarms. The ability to predict falls in patients by tracking and modelling their behaviour has received little research. This section offers a thorough analysis of the research on fall detection, highlighting numerous solutions from various angles. The methods, datasets, and advantages/disadvantages of this research are summarised in Table 1.

**TABLE 1:** Research gap.

Author	Dataset	Advantage	Methodology	Disadvantage
Musci et al. (2018)	SisFall	Prevent a senior from lying helpless for hours or days	Fall Detection System. (FDS)	Blind spots, which are owing to the presence of a single camera, and the occlusion problem because of a dynamic background
Al-Rakhami et al. (2021)	SMARTWATCH and SMARTFALL	“The proposed fall detection framework combines DL algorithms and mobile edge computing in 5G wireless networks, resulting in improved accuracy for IoMT-based healthcare applications”	“deep gated recurrent unit (DGRU) neural network”	“While the framework achieves higher accuracy rates, its reliance on DL algorithms requires a significant amount of data and processing power.”
Taramasco et al. (2018)	Real time data	It enables multi-tasking and eases the workload for existing resources. Operates 24 x 7 without interruption or breaks and has no downtime	Artificial intelligence (AI)	The rapid progress of AI has raised several concerns that 1 day, AI will grow uncontrollably, and eventually wipe out humanity
Martínez-Villaseñor et al. (2019)	UP-Fall Detection Dataset	“It keeps a check on the routers, firewalls, key servers, and files and uses its database to raise the alarm and send notifications.”	UP-Fall Detection Dataset	Inconvenience due to wearing/attaching the sensor on a part of the body
Hassan et al. (2019)	MobiAct	To alert when a fall event has occurred	Mobile-enabled fall detection (MEFD)	Increased false alarms

In 2022, Yang et al. (2022) proposed a highly accurate, fast real-time fall detection technology that can protect user privacy. It uses infrared array sensors. Additionally, it was advised to carry out a feature fusion following the extraction of the four-dimensional properties of centroid change, speed, area change, and variance change from the temperature data captured by the infrared array sensor. Şengül et al. (2022) developed a mobile application that gathers accelerometer and gyroscope data from smartwatches for the purpose of detecting falls in the medical field. To appropriately identify activities, the gathered data was transmitted to the cloud to be processed using a deep learning method, especially the bi-directional long short-term memory (BiLSTM) neural network.

In 2021, Wang et al. (2021) used system named FallViewer has been proposed that examines the channel state information (CSI) of Wi-Fi signals. To acquire fine-grained data for deviation correction, phase and amplitude calibration approaches were described. An antenna power modulation technique was created to prevent multipath interference. In order to increase FallViewer’s ability to adapt to various settings, a double sliding window was also used to create a variable threshold. After extracting characteristics from the cleaned-up Wi-Fi signal, FallViewer used LibSVM for categorization. Waheed et al. (2021) developed a reliable, noise-resistant fall detection system (FDS) that can still function with missing data. Wearable sensors and Deep Learning, specifically Recurrent Neural Networks (RNNs) with a Bidirectional Long Short-Term Memory (BiLSTM) stack, are used to implement the FDS. Vishnu et al. (2021) proposed a technique for modelling the spatiotemporal elements of human fall detection using fall motion vectors. The technique produces a Gaussian mixture model (GMM) known as a fall motion mixture model (FMMM) using the histogram of optical flow and motion boundary histogram characteristics. This FMMM captures motion properties in both fall and non-fall movies, producing a high-dimensional representation. Zurbuchen et al. (2021) presented an expansion of our earlier work on creating an FDS that makes use of an at-the-waist inertial measurement device. The SisFall dataset, which contains information on falls and activities of daily living, is used in the study. Techniques for pre-processing and feature extraction were used, after which five Machine Learning algorithms’ performance was compared.

In 2019, Martínez-Villaseñor et al. (2019) presented dataset for detecting upfalls. The dataset consists of raw data and feature sets that were gathered from 17 young, healthy persons who underwent 11 activities and three falls and were free of any impairments. The collection also gathers information from vision devices, ambient sensors, and wearable sensors totalling more than 850 GB. Van Thanh et al. (2019) proposed to develop a low-cost fall detection system that is capable of precisely tracking elderly people’s falls. The fall identification algorithm compares the acceleration with the lower fall threshold and upper fall threshold values in order to precisely identify a fall event. Our device will promptly send the contacts’ whereabouts via SMS and voice calls in the case of a fall. An app for a smartphone will ensure that the notifications are delivered to the elderly person’s family members so that immediate medical aid can be provided. Hassan et al. (2019) introduced the MEFD framework, which can identify senior falls and aid family members and carers by quickly locating them. The system uses a wireless access point in the house to send outside SMS warnings to a hospital or carer using a mobile network base station or inside sound alerts to family members using a mobile access point.

## 3 Proposed methodology

**FIGURE 1:**
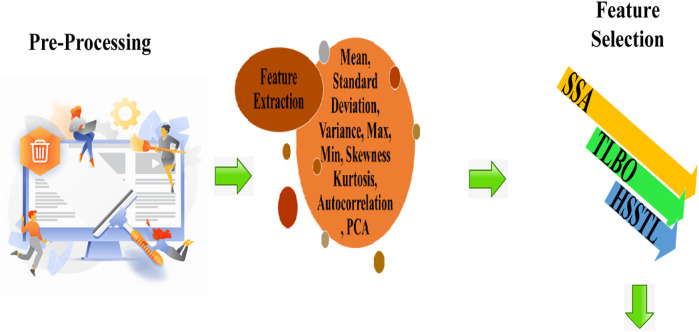
Overall proposed architecture.

**FIGURE 2:**
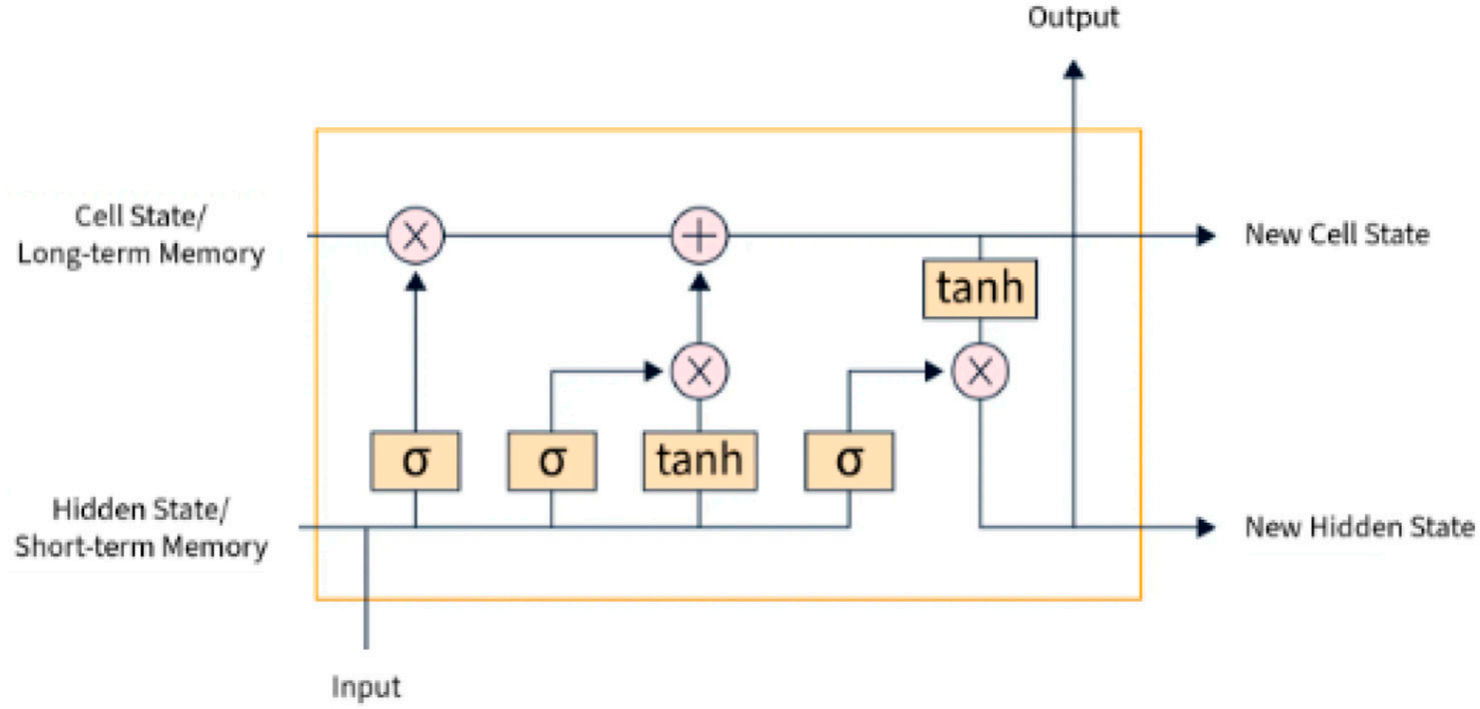
LSTM.

**FIGURE 3:**
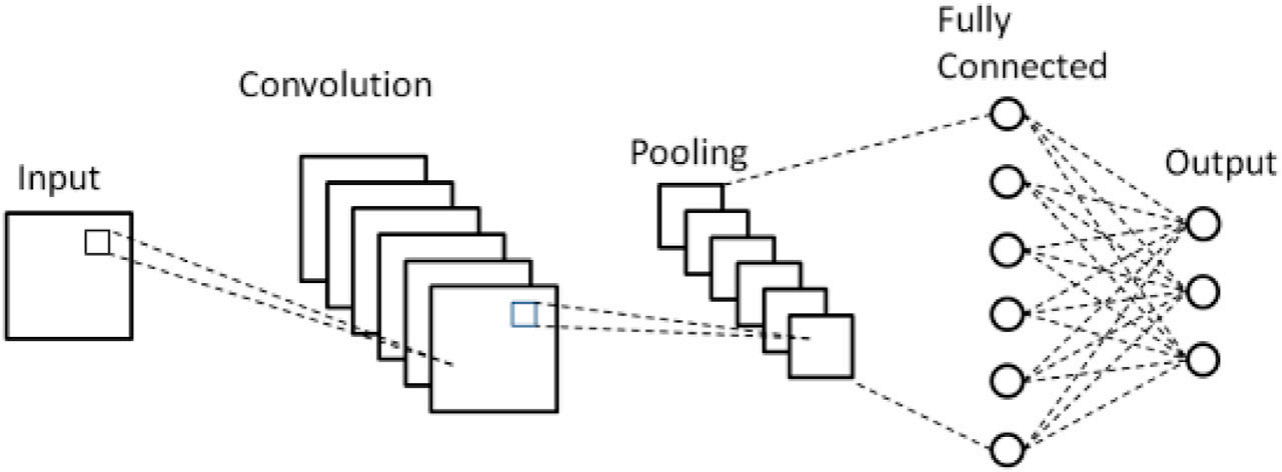
CNN.

**FIGURE 4:**
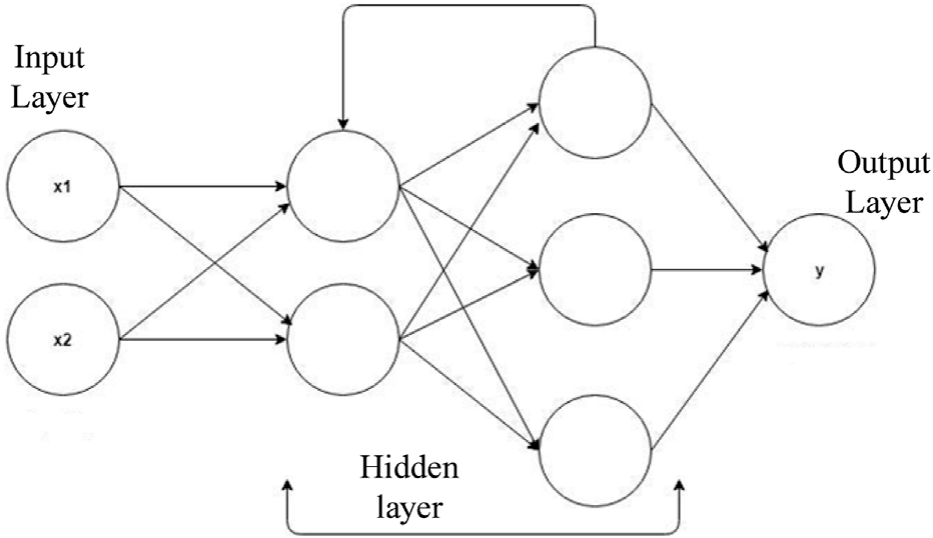
RNN.

In response to the increasing population of elderly individuals and the growing concern for their safety, a novel approach is proposed that utilizes Blockchain and IOT technology to develop smart and secure virtual assistants for fall detection in elders. The system employs a combination of LSTM, optimized CNN, and RNN models to achieve accurate fall detection. Additionally, a hybrid optimization model is introduced, integrating SSA and TLBO, to optimize the weights of the CNN. This approach aims to improve the accuracy and efficiency of fall detection, ultimately ensuring the safety and wellbeing of elderly individuals. Data collection, feature extraction, feature selection, fall detection using TriNet, and emergency response and assistance are the five main steps of the proposed model. Figure 1 shows the overall proposed architecture. Figures 2–4 shows the architecture of CNN, LSTM and RNN.

### 3.1 Data acquisition

To address the requirement for precise fall prediction and detection among older persons, the Elderly Fall Prediction and Detection dataset (Kaggle, 2023a) was developed. While most wearables currently on the market only focus on fall detection, this dataset attempts to offer insights into foreseeing falls. The dataset introduces the cStick, a revolutionary device that can help older persons who are hard of hearing or visually challenged by keeping track of falls and estimating their likelihood. The cStick device has a number of sensors that can record important information about falls and their forecast. These variables include the following: the distance travelled, the pressure (low, medium, and high) levels, the heart rate variability (HRV), the blood sugar levels, the oxygen saturation levels (SpO2), and the accelerometer readings. Each test offers valuable information regarding the physiological state of the subject and their immediate surroundings. In addition, the dataset divides the falls into three groups according to the evaluation of the cStick device. The classifications are: no fall detected, individual slipped, stumbled, or predicted fall, and actual fall. A thorough comprehension of the fall episodes is made possible by this classification, which also makes it possible to make precise predictions and identify slips and falls. 2039 data points altogether make up the provided dataset, which has been split into training and testing sets based on various learning rates. 1,427 data points are set aside for training and 612 for testing, assuming a learning rate of 70%. 1,631 data points are used for training with an 80% learning rate, while 408 data points are set aside for testing. Following are the labels’ class distributions across datasets: “No Fall” (690 occurrences), “Slip” (682 occurrences), and “Fall” (667 occurrences).

### 3.2 Pre-processing

In this research work, pre-process is carried out using Missing Value Removal, and Null Value Handling.

#### 3.2.1 Missing value removal

Preprocessing is essential for ensuring that fall detection systems for the safety of the elderly are accurate and reliable. The handling of missing values in the data is an essential part of preprocessing. Missing values can occur for a number of reasons, including sensor problems, transmission problems or lack of data collection. It is crucial to properly handle this missing information in order to produce robust research. In the preprocessing step of fall detection for elderly safety, there are numerous methods to take into consideration for missing value removal. One easy technique is to remove all rows with missing values. However, if the missing values are not spread randomly, then using this method could lead to the loss of important data. When there is little missing data and deleting a few rows will not have a big impact on the data as a whole, rows should be deleted.

#### 3.2.2 Null value handling

Fall detection systems utilize various sensors and devices to monitor the movements and activities of the elderly individuals, aiming to detect and alert caregivers or emergency services in the event of a fall. During the data collection process, it is common to encounter missing or null values due to various reasons such as sensor malfunction, data transmission errors, or absence of relevant data.

### 3.3 Feature extraction

In this research work, features are extracted using statistical features, autocorrelation, and PCA. The chosen feature extraction methods–statistical features, autocorrelation, and PCA–are integral due to their ability to capture nuanced movement patterns in sensor data. Statistical features encapsulate data distribution, aiding anomaly detection during falls. Autocorrelation identifies rhythmic deviations, significant for fall disruption. PCA reduces dimensionality, accentuating relevant variation. Collectively, these methods enrich the model’s understanding of motion dynamics, enhancing fall detection accuracy by discerning normal activities from fall-related anomalies.

#### 3.3.1 Statistical features

In this study, standard deviation, mean, variance, minimum, maximum, skewness, and kurtosis of the data are extracted. These characteristics offer important details regarding the distribution, central tendency, variability, and shape of the data.

##### 3.3.1.1 Mean

Mean is defined as the total number of elements divided by the total number of elements in a set. Calculating the mean gives us a good understanding of the entire set of data. Consequently, the mean formula is calculated as per Eqs 1, 2.

$$Mean=\frac{Sum\;of\;all\;the\;elements}{Number\;of\;elements}$$ (1)

$$\bar{D}=\frac{\sum d}{n}$$ (2)

Where, $\bar{D}$ = mean value, d = Items given, n = Total number of items

##### 3.3.1.2 Standard deviation

The variance of the data is square-rooted to produce the standard deviation as shown in Eq. 3. “The standard deviation is a measure of variance from the mean that takes spread, dispersion, and spread into account. The standard deviation displays an ordinary deviation from the mean. It is a well-liked measure of variability since it utilises the original units of measurement from the data set”.

$$SD\;\left(\sigma \right)=\sqrt{\frac{\sum {\left(y_{i}-\mu \right)}^{2}}{N}}$$ (3)

##### 3.3.1.3 Variance

The variance of a data set is the measure of numerical variation. Variance, in particular, determines how far off each integer in the set is from the mean and, consequently, from the other numbers in the set. This is mathematically shown in Eq. 4.

$$Variance=\frac{\sum {\left(r^{pre}-\mu \right)}^{2}}{X}$$ (4)

##### 3.3.1.4 Maximum

Maximum value the highest value observed in a given dataset or a specific subset of data, the maximum value provides information about the upper limit or peak of a particular variable or signal. When extracting features from a dataset, the maximum value can be calculated for individual data points, time windows, or specific segments of interest.

##### 3.3.1.5 Minimum

The minimum value represents the lowest value observed in a given dataset or a specific subset of data, the minimum value provides information about the lower limit or nadir of a particular variable or signal. When extracting features from a dataset, the minimum value can be calculated for individual data points, time windows, or specific segments of interest.

##### 3.3.1.6 Skewness

A measure of a distribution’s symmetry is its skewness. Actually, calling it a measure of asymmetry would be more appropriate. A typical normal distribution has zero skew and is entirely symmetrical as shown in Eq. 5.

$$Skewnes=\frac{3\left(Mea-Media\right)}{StandardDeviation}$$ (5)

##### 3.3.1.7 Kurtosis

“Kurtosis is a measurement that shows how heavily or thinly the data are distributed in comparison to a normal distribution. To put it another way, data sets with a high kurtosis are more likely to have huge outliers or heavy tails. Data sets with low kurtosis usually have light tails or no outliers”. This is mathematically shown in Eq. 6.

$$Kurtosi\;\left(high\;order\right)=\frac{4^{th}Momen}{{4^{th}Momen}^{2}}$$ (6)

#### 3.3.2 Autocorrelation

Autocorrelation is a widely used mathematical operation in the time domain that quantifies the similarity of a given time signal to itself over a time scale. The autocorrelation of a time series, denoted as $au\left(n\right),$ can be computed using Eq. 7.

$$s_{cs}\left(m\right)=\left\{\begin{array}{l} \sum_{i=0}^{I-m-1}{au}_{i+m}{au}_{i}\;m\geq 0\\ s_{cs}\left(-m\right)\;m<0 \end{array}\right.$$ (7)

As per Eq. 9, the subscript cs represents the correlation sequences, and the lag factor of the autocorrelation process, denoted by m , signifies the time shift parameter. For a time, series with I finite data points, the resulting autocorrelation sequence comprises ( 2I−1 ) data points. In this study, autocorrelation is utilized to measure the similarity of vibration signals in the time domain.

#### 3.3.3 Principal Component Analysis

PCA can be used as a feature extraction technique in a fall detection system for elderly safety. By applying PCA to the dataset containing various correlated variables related to fall detection, the dimensionality of the data is reduced while retaining the most important information.

1. Normalize the pre-processed data using min-max normalization and is represented as per Eq. 8.

$$X_{norm}=\left(\frac{x-\mu }{\sigma }\right)$$ (8)

2. Compute the covariance matrix and given as per Eq. 9.

$$c=\frac{\left(x_{norm}-\mu \right){\left(x_{norm}-\mu \right)}^{T}}{N-1}$$ (9)

Where X is the pre-processed data matrix, μ is the mean vector of $x_{norm}$ , and N is the count of samples.

3. Eigen Decomposition

To compute the eigenvalues and eigenvectors of matrix C , sort the eigenvalues in ascending order, these steps are followed.

o Compute the eigenvalues and eigenvectors of matrix C .
o Sort the eigenvalues in ascending order.
o determine the optimal value of k , the count of principal components.
o Select the top k eigenvalues.

4. Eigenvalue Thresholding

Define thresholding value $\lambda_{eig}$ and threshold eigen vector $V_{vec}$ to separate eigen value from noise, discard eigen value small than $\lambda_{eig}$ . In eigenvalue thresholding, a threshold value $\lambda_{eig}$ is chosen to distinguish between significant eigenvalues and noise. Eigenvalues smaller than $\lambda_{eig}$ , along with their corresponding eigenvectors, are discarded, while retaining the significant eigenvalues and eigenvectors $V_{vec}$ . This process effectively separates the eigenvalues from noise, improving the representation of the underlying data structure and given as per Eqs 10, 11.

$$\lambda_{select}=\left[\lambda_{i},if\lambda_{i}\geq \lambda_{eig};0,if\;\lambda_{i}<\lambda_{eig}\right]$$ (10)

$$V_{select}=\left[V_{i},ifV_{i}\geq V_{vec};0,if\;V_{i}<V_{vec}\right]$$ (11)

Where $\lambda_{select}$ is the selected eigen values and $V_{select}$ is the selected eigen vector.

Retained eigen values $\lambda_{select}$ and $V_{select}$ are used for feature extraction and represented as per Eq. 12.

$$TF=D^{T}*V_{select}$$ (12)

Where $D^{T}$ represents the transposed data matrix, and TF is the transformed feature matrix. The extracted features are passed as input to the feature selection.

### 3.4 Feature selection

The proposed hybrid HSSTL technique combines (TLBO) and (SSA) for feature selection. TLBO’s global exploration and SSA’s localized search enhance the identification of relevant features, addressing the challenge of identifying critical fall-related attributes. This contribution ensures the most informative features are chosen, improving system accuracy. To further refine the feature set obtained through PCA, a feature selection mechanism can be applied to identify the most optimal features. In this case, a hybrid optimization model can be utilized for feature selection. The integration of the hybrid HSSTL technique, which merges (TLBO) with (SSA) for feature selection, is a deliberate choice rooted in the desire to leverage the distinctive strengths of both algorithms. TLBO excels in its global exploration capabilities, while SSA’s localized search behavior allows for fine-tuning around potential solutions. This blend aims to strike an optimal balance between exploration and exploitation, enhancing the feature selection process’s efficiency and effectiveness. The HSSTL hybrid approach introduces, however, a degree of complexity and requires parameter tuning. In comparison to conventional methods like (RFE) or (GA), the HSSTL approach holds promise in uncovering intricate inter-feature relationships, potentially leading to improved fall detection models. Nonetheless, thorough empirical validation across diverse datasets is essential to ascertain its robustness and superiority over existing techniques.

#### 3.4.1 Hybrid teaching-learning spring search (HTLSS)

The Teacher Phase and the Learner Phase are the two stages of the TLBO method. The teacher, who is the expert in the community, shares knowledge with the students during the teacher phase. The teacher’s objective is to raise the class’s average performance and knowledge level. Interaction between learners occurs during the learner phase, and they benefit from one another’s knowledge and experiences. Their interaction improves their output and all-around performance. A group of learners are modelled by the population in the population-based optimisation technique known as TLBO. The outcomes of the learners are comparable to fitness value in other optimisation methods. The TLBO process uses instructor direction and student engagement to incrementally raise learners’ mean performance in order to reach a global solution. SSA is an optimization algorithm that simulates an artificial system using the principles of spring force. It defines an initial configuration of objects within the problem space and iteratively updates their positions based on the governing laws and objective function. The algorithm continues until a stopping criterion is met, aiming to find the optimal solution within the problem domain. HTLSS combines the population-based learning of TLBO with the spring force dynamics of SSA. It leverages TLBO’s teacher-student interaction and knowledge dissemination with SSA’s spring-based information conveyance. This hybrid approach aims to enhance the optimization performance by incorporating the strengths of both algorithms.

Step 1: Population Initialization: The number of students or population size in the population initialization stage of the optimisation process. A condition known as the stopping criterion establishes when the algorithm should stop iterating. Its purpose is to stop the algorithm from running endlessly. Generate random opposition-based learning solutions for each individual in the population.

$${\hat{x}}_{j}={lo}_{j}-{up}_{j}-rand*x_{j}$$ (13)

As per Eq. 13, ${\hat{x}}_{j}$ represents the opposite solution, $x_{j}$ is the current solution, ${lo}_{j}$ and ${up}_{j}$ define the lower and upper bounds respectively.

Step 2: Compute the fitness value for each individual in the population based on the defined fitness function as per Eq. 14.

$$Fit=\min\left(e\right)$$ (14)

Step 3: Sort the solutions in ascending order based on the computed fitness values. The first best solution $f\left(x_{best}\right)$ becomes the chief teacher, while the remaining four best solutions are assigned as other teachers, ranked according to the performance of the best teacher as given by Eqs 15–17.

$$f{\left(x_{teacher}\right)}_{1}=f\left(x_{1}\right),wheref\left(x_{1}\right)=f\left(x_{best}\right)$$ (15)

$$f\left(x_{s}\right)=f\left(x_{1}\right)-{rand}^{*},s=2,3,4,5$$ (16)

$${\left(x_{teacher}\right)}_{s}=f{\left(x\right)}_{s}$$ (17)

If $f\left(x_{1}\right)\geq f\left(x_{l}\right)>f\left(x_{2}\right),$

The learners are assigned to the first teacher (chief teacher) for instruction and guidance (i.e., $f\left(x_{1}\right)$ ). Subtract the mean learner result from the instructor result for each subject to calculate the difference between the teacher result and the mean learner result for each subject. The resulting difference indicates how the teacher’s performance compares to the average performance of the learners in each subject. At each iteration al , let $M_{al}$ represent the mean resultant of learners l in subject a and $T_{al}$ represent the teacher. The teacher $T_{al}$ aims to adjust the mean $M_{al}$ towards its own level, resulting in a new mean denoted as $x_{1}$ . As per Eqs 18, 19, it is given as,

$${diff\_mean}_{al}=q_{i}\left(x_{1}\right)-T_{f}M_{al}$$ (18)

$$x_{new\left(2\right)}=x_{old}+{diff\_mean}_{al}$$ (19)

Here, $T_{f}$ is a teaching factor that determines the extent of the mean adjustment, and $q_{i}$ defines the random-number in the range [0, 1]. $T_{f}=round\left[1+rand\left(0,1\right)\right].$

Step 4: Follow the strategy of pairing each learner with the instructor whose fitness value is closest to the learner’s fitness value to allocate learners to the teachers based on their fitness function.

$$For\;l=1:\left(n-s\right)$$

If

$$f\left(x_{2}\right)\geq f\left(x_{l}\right)>f\left(x_{3}\right)$$

Assign learns $f\left(x_{l}\right)$ to teacher 2, and update the position of the teacher.

$$x_{new\left(2\right)}=x_{old}+rand{\left(x_{teacher}\right)}_{2}-T_{f}.mean$$ (20)

In Eq. 20, $x_{new\left(2\right)}$ is the position of learner based on the teacher’s position $x_{2}$ , $x_{old}$ represents the old position of the learner, ${\left(x_{teacher}\right)}_{2}$ represents the position of the teacher (i.e., $f\left(x_{2}\right)$ ), and mean is the average count of search solution in the population.

Else.

Assign the learner $f\left(x_{l}\right)$ to teacher T.

End.

Step 5: Retain the best solution, or the elite solution, from each group.

Step 6: Calculate the mean results of each group of learners.

Step 7: Determine the difference between the current mean and the teacher’s related result for each group. Based on the adaptive teaching factor, this calculation.

Step 8: Update the knowledge of the learners in each group by incorporating the knowledge of the respective teachers as per Eqs 21, 22.

$$x_{new\left(1\right)}=x_{old\left(1\right)}+rand\left[\left(1-\frac{t}{T}\right)x_{old\left(1\right)}+\left(\frac{t}{T}\right)x_{teacher\left(1\right)}-x_{old\left(1\right)}\right]*T_{f}$$ (21)

$$x_{new\left(2\right)}=x_{old\left(2\right)}+rand\left[\left(1-\frac{t}{T}\right)x_{old\left(2\right)}+\left(\frac{t}{T}\right)x_{teacher\left(2\right)}-x_{old\left(2\right)}\right]*{diff_{mean}}_{al}$$ (22)

Step 9: Update the knowledge of the learners in each group by leveraging the knowledge and interaction among the other learners as per Eqs 23–25.

$$x_{new\left(1\right)}=x_{old\left(1\right)}+rand\left[\left(1-\frac{t}{T}\right)x_{new\left(1\right)}+\left(\frac{t}{T}\right)x_{teacher\left(1\right)}-e_{f}∙x_{old\left(1\right)}\right]$$ (23)

$$x_{new\left(2\right)}=x_{old\left(2\right)}+rand*\left(diff\_mean\right)+rand\left[x_{teacher\left(2\right)}-{e_{f}∙x}_{new\left(2\right)}\right]$$ (24)

$$x_{new\left(3\right)}=x_{old\left(3\right)}+rand\left[x_{teacher\left(2\right)}-x_{new\left(3\right)}\right]+rand\left[x_{teacher\left(3\right)}-{e_{f}∙x}_{new\left(3\right)}\right]$$ (25)

Where $e_{f}$ represents the exploration factor and $e_{f}=round\left(1+rand\right)$

Step 10: The elite solution, which stands for a highly appropriate solution replaces the worst decision within each group. This process aids in raising the general standard of each group’s solutions. The worst solutions are changed, then all the groups are joined with the modified solutions from each group. This makes it possible to explore the problem space more thoroughly. The algorithm’s outcome is the best result so far, taking into account all the groups. The ideal solution discovered through the optimisation process is represented by this solution. Finally, the algorithm is terminated. The structure of HTLSS pseudocode is shown in Algorithm 1.

Algorithm 1 HTLSS.

Initialize number of students/population, stopping criterion
Generate random opposition-based learning
Compute fitness as per Eq.
Sort solution in ascending order using Eqs 2, 3
Calculate mean using Eqs 5, 6
Assign learners to teachers using proposed Eq. 89
Identify the best solution
Calculate mean result of each group
Calculate difference between mean and result of group
Update knowledge of learners-based knowledge of teachers using Eqs 7, 8
Update knowledge of learners-based knowledge of other learners using proposed Eq. 7 and Eqs 9–11
Replace the worst solution
Combine all the groups
Return the best solution
End

### 3.5 Fall detection via TriNet

The integration of (LSTM), optimized (CNN), and (RNN) as TriNet creates a robust fall detection framework. LSTM captures temporal dependencies, optimized CNN extracts spatial information, and RNN models sequential patterns. These models collectively address the complexity of fall detection, improving accuracy. The optimal features are selected and the output from feature selection is passed as an input to the detection phase. TriNet, a fall detection system for elderly safety, integrates a combination of LSTM, optimized CNN, and RNN models to achieve accurate fall detection. The fall detection model, TriNet, comprises a synergistic fusion of (LSTM), optimized (CNN), and (RNN) architectures, strategically designed to capture temporal and spatial patterns inherent in sensor data. The LSTM component handles sequential dependencies, crucial for detecting falls characterized by sudden changes. The optimized CNN extracts hierarchical features from sensor data, identifying relevant patterns and enhancing model discriminability. RNN augments the temporal context, further refining fall detection accuracy. Hyperparameters, including learning rates, batch sizes, and optimizer settings, are meticulously tuned to strike a balance between convergence and generalization. The training process involves forward and backward propagation, iteratively refining weight parameters. The model’s performance is validated through rigorous testing, ensuring its efficacy in accurately identifying fall incidents among elderly individuals.

#### 3.5.1 LSTM

A form of RNN architecture called LSTM is created to address the vanishing and exploding gradient problem, which is a typical problem in conventional RNNs. LSTMs are excellent for processing time-series data because they can identify long-term dependencies in sequential data. Information can be stored in an LSTM cell’s memory and converted from input to output while the cell is in operation. An LSTM cell is made up of the input gate, update gate, forget gate, and output gate. The input gate, as the system’s name suggests, chooses which information the neuron will process, the update gate changes the cell, and the output gate creates new long-term memory. When the LSTM absorbs long-term memory, short-term memory, and the input sequence at one time step and develops new long-term memory, short-term memory, and new output sequence at another time step, these four essential LSTM components will function and interact in a unique way. Which data must be delivered to the cell is decided by the input gate, which is mathematically described in Eq. 26.

$$i_{t}=\sigma \left(w_{i}*\left[h_{t-1},X_{t}\right]+b_{i}\right)$$ (26)

The operator * multiplies each element of the vectors individually. The forget gate regulates which information from the previous memory is to be disregarded, and it is mathematically characterised by Eq. 27.

$$f_{t}=\sigma \left(w_{f}*\left[h_{t-1},X_{t}\right]+b_{f}\right)$$ (27)

The update gate, represented theoretically as per Eqs 28, 29, modifies the cell state.

$$\tilde{c_{t}}=\tan h\left(w_{C}*\left[h_{t-1},X_{t}\right]+b_{c}\right)$$ (28)

$$c_{t}=f_{t}*C_{t-1}+i_{t}*\;\tilde{c_{t}}$$ (29)

Eqs 30, 31 is also capable of updating the output as it is supplied by the previous time step.

$$O_{t}=\sigma \left(w_{o}*\left[h_{t-1},X_{t}\right]+b_{o}\right)$$ (30)

$$h_{t}=O_{t}*\tan h\left(C_{t}\right)$$ (31)

#### 3.5.2 Optimized CNN

Due to its ability to automatically extract spatial characteristics, CNN, “an Artificial Neural Network (ANN) based on deep learning theory, has found widespread usage in the field of detecting geriatric falls. The activation function, convolutional layer, pooling layer, and fully connected layer are the four primary layers that make up CNN. The proposed model optimizes CNN through HSSTL, fine-tuning its feature extraction capabilities. This addresses the challenge of feature representation in deep learning models, increasing the sensitivity to fall patterns and reducing false positives. HSSTL’s hybrid approach enhances CNN’s performance, contributing to improved accuracy.

##### 3.5.2.1 Convolutional layer

The matrix multiplication function in the traditional neural network is replaced by the convolution operation in the convolutional layer, which is utilised to extract picture information and learn the mapping between the input and output layers. Sharing parameters during the convolution operation enables the network to learn just one set of parameters, significantly cutting down on the number of parameters and dramatically improving computing efficiency. A convolution operation is defined as in Eq. 32.

$$f_{j,g}=\sum_{i=o}^{h}\sum_{l=0}^{h}k_{i,l\;m_{j+i,}g+l}$$ (32)

where $k_{i,l}$ is the weight of convolutional kernel at mandl ; $m_{j+i,}$ is the pixel value of image at i and g ; h is the height and width of convolutional kernel.

##### 3.5.2.2 Activation function

In order to avoid vanishing gradients and hasten training, CNN typically uses Rectified Linear Unit (ReLU) activation functions. Equation 33 provides a description of ReLU’s goal.

$$ReLU\left(n\right)=\left\{\begin{array}{c} f\;f>0\\ 0\;f\leq 0 \end{array}\right.$$ (33)

##### 3.5.2.3 Pooling layer

The network’s computational complexity can be reduced by the pooling layer, which also concentrates the data into feature maps. Max pooling is a common pooling layer shown in Eq. 34.

$$MxPl\left(o_{ot},k_{ot}\right)=\left\{\begin{array}{c} o_{ot}=floor\left(\frac{\left(o_{g}+2q-p\right)}{h}+1\right)\\ k_{ot}=floor\left(\frac{\left(e_{g}+2q-p\right)}{h}+1\right) \end{array}\right.$$ (34)

where $floor\left(m\right)$ represents round up function, $o_{ot}$ is the output height, $k_{ot}$ is the output width, $o_{ot}$ is the input height, $k_{g}$ is the input width, q is the padding, p is the kernel size, h is the kernel stride”.

#### 3.5.3 RNN

Recurrent neural networks (RNNs), a type of artificial neural network, are designed for processing sequential data, including time series and natural language data. RNNs preserve a hidden state to detect temporal correlations between inputs. They solve the vanishing gradient problem by employing gates, which allow them to recall or forget information. A chain of memory cells makes up the RNN architecture. Input, output, and forget gates are present in each cell. The output gate decides the current state output, the forget gate regulates the retention of prior states, and the input gate receives fresh input as in Eq. 35.

$$k_{t}^{\left(s\right)}=\sigma \left(u_{og}^{\left(s\right)}.\left[k_{t-1}^{\left(s\right)},x_{t}^{\left(s\right)}\right]+M_{r}^{\left(s\right)}k_{t-1}^{\left(s\right)}+b_{og}^{\left(s\right)}\right)$$ (35)

Limited representation results from simple RNNs’ inability to understand information from nodes further along the sequence. In order to control information flow and solve the issue of long-term stability, LSTM employs gates. The forward propagation process of the LSTM is expressed in Eqs 36–41. The LSTM cell state is made up of two parts: freshly learned information scaled by the input gate, and long-term memory from earlier moments preserved by the forget gate.

$${ie}_{t}^{\left(n\right)}=\sigma \left(u_{ie}^{\left(n\right)}.\left[a_{t-1}^{\left(n\right)},x_{t}^{\left(n\right)}\right]+b_{ie}^{\left(n\right)}\right)$$ (36)

$${fo}_{t}^{\left(n\right)}=\sigma \left(u_{fo}^{\left(n\right)}.\left[a_{t-1}^{\left(n\right)},x_{t}^{\left(n\right)}\right]+b_{fo}^{\left(n\right)}\right)$$ (37)

$${og}_{t}^{\left(n\right)}=\sigma \left(u_{og}^{\left(n\right)}.\left[a_{t-1}^{\left(n\right)},x_{t}^{\left(n\right)}\right]+b_{og}^{\left(m\right)}\right)$$ (38)

$$h_{t}^{\left(n\right)}=o_{t}^{\left(n\right)}.\tanh\left(L_{l}\right)$$ (39)

$${{\tilde{i}}_{t}}^{\left(n\right)}=\tanh\left(u_{i}^{\left(n\right)}.\left[a_{t-1}^{\left(n\right)},x_{t}^{\left(n\right)}\right]+b_{i}^{\left(n\right)}\right)$$ (40)

$$i_{t}^{\left(n\right)}=f_{t}^{\left(n\right)}.i_{t-1}^{\left(n\right)}+e_{t}^{\left(n\right)}.{{\tilde{i}}_{t}}^{\left(n\right)}$$ (41)

Here ${ie}_{t}^{\left(n\right)}$ , ${og}_{t}^{\left(n\right)}$ , ${fo}_{t}^{\left(n\right)}$ and $i_{t}^{\left(n\right)}$ are the input, output gate, forgotten, and cell state of the *n*th RNN layer at time l , respectively; $u_{ie}^{\left(n\right)}$ , $u_{fo}^{\left(n\right)}$ , $u_{og}^{\left(n\right)}$ , $u_{i}^{\left(n\right)}$ are all weight coefficient matrices; and $b_{ie}^{\left(n\right)}$ , $b_{fo}^{\left(n\right)}$ , $b_{og}^{\left(m\right)}$ and $b_{i}^{\left(n\right)}$ are all bias vectors.

In comparison to existing fall detection solutions, the TriNet system offers distinct advantages. TriNet’s fusion of LSTM, optimized CNN, and RNN empowers robust fall detection, surpassing traditional methods. Its hybrid feature selection approach optimizes accuracy by integrating TLBO and SSA. Unlike single-method systems, TriNet’s multi-metric evaluation enhances reliability in diverse scenarios. Moreover, its integration with blockchain ensures secure data storage and swift alerts, setting it apart in terms of accuracy, response times, and user-friendliness.

### 3.6 Emergency response and assistance

IoT sensors capture movement data from seniors, encrypted and transmitted to the blockchain. Immutable and decentralized, the blockchain securely stores data, enhancing fall detection reliability. The integration ensures transparent, tamper-proof data flow, bolstering TriNet’s credibility and robustness in detecting elderly falls. Leveraging blockchain technology ensures secure and tamper-proof storage of fall detection data. Blockchain addresses concerns of data privacy, integrity, and accountability, providing a trustworthy platform for storing sensitive information. This contribution enhances the system’s reliability and trustworthiness.

After detecting a fall, the system takes the following actions. The information relating to fall detection and other related data is safely stored on the Blockchain network. Blockchain technology ensures the integrity, immutability, and tamper resistance of the stored data, providing a reliable record of the fall incidence. The Blockchain network is used to communicate notifications about the fall incidence to approved emergency care providers, neighbours, or family members. The notifications may include details regarding the location of the fall, the injured individual, and any other relevant information that was gathered by the wearable technology or fall detection system. The seriousness of the fall incident influences how it should be addressed, which may be done by taking into account a variety of factors. If the system determines that the fall was severe or if the victim is not responding, it can immediately request medical assistance. This may require contacting emergency medical services or an ambulance to provide immediate medical assistance. The system can contact emergency services to notify them of the occurrence and provide them with the information they need to respond if the fall is deemed substantial but not immediately life-threatening. The device can also alert nearby family members, neighbours, or carers who can provide immediate assistance. This can be done by sending direct notifications to their devices or by setting off alarms close to the fall incident. Different emergency response and assistance protocols may be utilised, depending on the system’s architecture, local legislation, and available resources. The goal is to provide a timely and appropriate response to the fall incident, placing the security and health of the affected individual first. The incorporation of a Blockchain network for secure fall detection data storage introduces intriguing possibilities, but it also demands careful consideration of data privacy and integrity. The model’s design takes into account potential security concerns through a series of measures. Data encryption prior to storage safeguards against unauthorized access, while utilizing a private or permissioned Blockchain restricts data visibility to authorized participants. The immutability of the Blockchain ensures data integrity. However, potential vulnerabilities, such as attacks and data leakage, are addressed through consensus mechanisms and access controls. Furthermore, smart contract vulnerabilities are addressed through rigorous auditing. Data linkage concerns are mitigated using techniques like zero-knowledge proofs. By embracing these measures, the proposed model aims to establish a robust and secure environment for the storage of fall detection information, preserving both privacy and integrity.

Acknowledging potential limitations, sensor accuracy directly influences fall detection precision. Balancing false positives and negatives poses a challenge, impacting user trust and system effectiveness. Additionally, real-time implementation with blockchain might face technical constraints, like transaction speed. Addressing these aspects ensures a comprehensive understanding of the proposed system’s scope and applicability.

## 4 Result and discussion

Python was used to implement the suggested model. The performance of the proposed method is analysed, and its results are compared to those of other algorithms, such as Teaching-Learning-Based Optimisation (TLO), Spring Search Algorithm (SSA), Support Vector Machine (SVM), and Convolutional Neural Network (CNN). Evaluation of the suggested model’s efficacy in terms of NPV, FPR, FNR, AND MCC, as well as accuracy, precision, recall, sensitivity, and specificity. In order to learn more and comprehend the properties of the dataset, exploratory data analysis (EDA) was carried out as part of the research paper. This included looking at the variability of the variables, spotting patterns, spotting outliers, and investigating correlations between various aspects. EDA served as a foundation for additional analysis and aided in the selection of data preparation and modelling strategies.

### 4.1 Performance metrics

“The performance is compared using the confusion matrix like accuracy, precision, sensitivity, specificity, f-measure, NPV, FPR, FNR, AND MCC. The formula for calculating the metrics is discussed in this section.

i. Accuracy

Accuracy is calculated as the fraction of correctly predicted cases to all examples.

$$Accuracy=\frac{TP+TN}{TP+FP+FN+TN}$$

ii. Precision

Precision is a valuable indication of how exactly the positive compounds are expected since it measures the percentage of properly anticipated positive instances to all test findings.

$$Precision=\frac{TP}{TP+FP}$$

iii. Sensitivity

The sensitivity value may be obtained by disunion the total positives by the proportion of true positive forecasts.

$$Sensitivity=\frac{TP}{TP+FN}$$

iv. Specificity

Specificity is defined as the proportion of accurately anticipated negative outcomes over all negative outcomes.

$$Specificity=\frac{TN}{TN+FP}$$

v. F_Measure

The F-Measure number strikes a balance between ensuring that each class only includes a single type of data item and fully identifying all data bits.

$$F\_Score=\frac{Presision.\;Recall}{Presision+Recall}$$

vi. Matthew’s correlation coefficient (MCC)

MCC is a two-by-two binary variable association measure, which is represented below,

$$MCC=\frac{\left(TP\times TN-FP\times FN\right)}{\sqrt{\left(TP+FN\right)\left(TN+FP\right)\left(TN+FN\right)\left(TP+FP\right)}}$$

vii. Negative Prediction Value (NPV)

A diagnostic test’s or another quantitative metric’s performance is described by NPV.

$$NPV=\frac{TN}{TN+FN}$$

viii. False Positive Ratio (FPR)

The false positive rate is deliberate by segmentation the total number of negative events by the number of negative events that were wrongly labelled as positive (false positives).

$$FPR=\frac{FP}{FP+TN}$$

ix. False Negative Ratio (FNR)

The false-negative rate, often known as the “miss rate,” is the probability that the test may fail to detect a real positive”.

$$FNR=\frac{FN}{FN+TP}$$

### 4.2 Overall performance analysis

The proposed and existing models’ performance measures, which both employ a 70% learning rate, are discussed in Table 2. The suggested model received the best accuracy score, 0.955679, demonstrating its excellent level of overall classification accuracy. TLO, SSA, SVM, and CNN were among the current models that performed well, with accuracies ranging from 0.886162 to 0.948079. With a precision score of 0.947812, the suggested model outperformed TLO (0.965592) and SSA (0.940286). Lower precision scores of 0.848839 and 0.866626 were obtained using SVM and CNN, respectively. With a sensitivity of 0.963715, the suggested model was the most sensitive, closely followed by CNN (0.950962) and SSA (0.956063). The sensitivity ratings for TLO and SVM were 0.871625 and 0.931444, respectively. The proposed model achieved a specificity score of 0.947749, comparable to the precision score. The existing models showed varying levels of specificity, with TLO having highest value of 0.967999, followed by SSA (0.940202) and CNN (0.859112). SVM exhibited the lowest specificity score of 0.841480. The proposed model achieved an F-Measure score of 0.925902, indicating a good balance between precision and recall. The existing models achieved F-Measure scores ranging from 0.859521 to 0.918549. The proposed model obtained an MCC score of 0.923724, indicating a strong correlation between predicted and actual labels. The existing models achieved MCC scores ranging from 0.820098 to 0.910007. (NPV), which measures the proportion of true negative predictions among all negative predictions, ranged from 0.811910 to 0.885927 across the models. The models showed FPR values ranging from 0.019067 to 0.026348 and FNR values ranging from 0.003342 to 0.006617.

**TABLE 2:** Proposed and existing model performance comparison: 70% learning rate.

Metrics	TLO	SSA	SVM	CNN	Proposed
Accuracy	0.920133	0.948079	0.886162	0.904731	0.955679
Precision	0.965592	0.940286	0.848839	0.866626	0.947812
Sensitivity	0.871625	0.956063	0.931444	0.950962	0.963715
Specificity	0.967999	0.940202	0.841480	0.859112	0.947749
F-Measure	0.889276	0.918549	0.859521	0.877531	0.925902
MCC	0.837701	0.910007	0.820098	0.837283	0.923724
NPV	0.811910	0.878872	0.855740	0.873672	0.885927
FPR	0.020208	0.026348	0.024195	0.024702	0.019067
FNR	0.004184	0.005642	0.006482	0.006617	0.003342

The performance comparison of the proposed and existing models with an 80% learning rate is summarized in Table 3. The proposed model achieved the highest accuracy of 0.974015, indicating its ability to classify with a high overall accuracy. The existing models, including TLO, SSA, SVM, and CNN, also performed well, with accuracies ranging from 0.875339 to 0.936500. SSA achieved the precision of 0.953798, followed closely by the proposed model with a precision score of 0.965998. TLO and CNN obtained moderate precision scores of 0.928802 and 0.856041, respectively, while SVM had the lowest precision of 0.838471. The proposed model demonstrated the highest sensitivity score of 0.982206, indicating its ability to correctly identify positive instances. TLO, SVM, and CNN also achieved relatively high sensitivity scores, ranging from 0.920068 to 0.944386. SSA exhibited a lower sensitivity score of 0.860979. The existing models showed varying levels of specificity. SSA achieved the highest specificity score of 0.956176, followed by the proposed model with a score of 0.965933. TLO, SVM, and CNN obtained specificity scores ranging from 0.831202 to 0.928718. The proposed model achieved a balanced F-Measure score of 0.943667. The existing models showed F-Measure scores ranging from 0.849023 to 0.907331. The proposed model exhibited a high MCC score of 0.971716, indicating a strong correlation between the predicted and actual labels. TLO, SVM, and CNN achieved lower MCC scores, while SSA had the lowest MCC score of 0.854074. NPV scores ranged from 0.827779 to 0.931955 across the models, with SVM achieving the highest NPV and SSA obtaining the lowest NPV. In terms of (FPR) and (FNR), the models showed FPR values ranging from 0.019634 to 0.026296 and FNR values ranging from 0.003441 to 0.006604.

**TABLE 3:** Proposed and existing model performance comparison: 80% learning rate.

Metrics	TLO	SSA	SVM	CNN	Proposed
Accuracy	0.936500	0.908895	0.875339	0.893681	0.974015
Precision	0.928802	0.953798	0.838471	0.856041	0.965998
Sensitivity	0.944386	0.860979	0.920068	0.939347	0.982206
Specificity	0.928718	0.956176	0.831202	0.848619	0.965933
F-Measure	0.907331	0.878414	0.849023	0.866813	0.943667
MCC	0.927794	0.854074	0.836127	0.853648	0.971716
NPV	0.896050	0.827779	0.872466	0.890748	0.931955
FPR	0.026296	0.020168	0.024147	0.024653	0.019634
FNR	0.005631	0.004176	0.006469	0.006604	0.003441

### 4.3 Overall Graphical Representation of performance analysis

**FIGURE 5:**
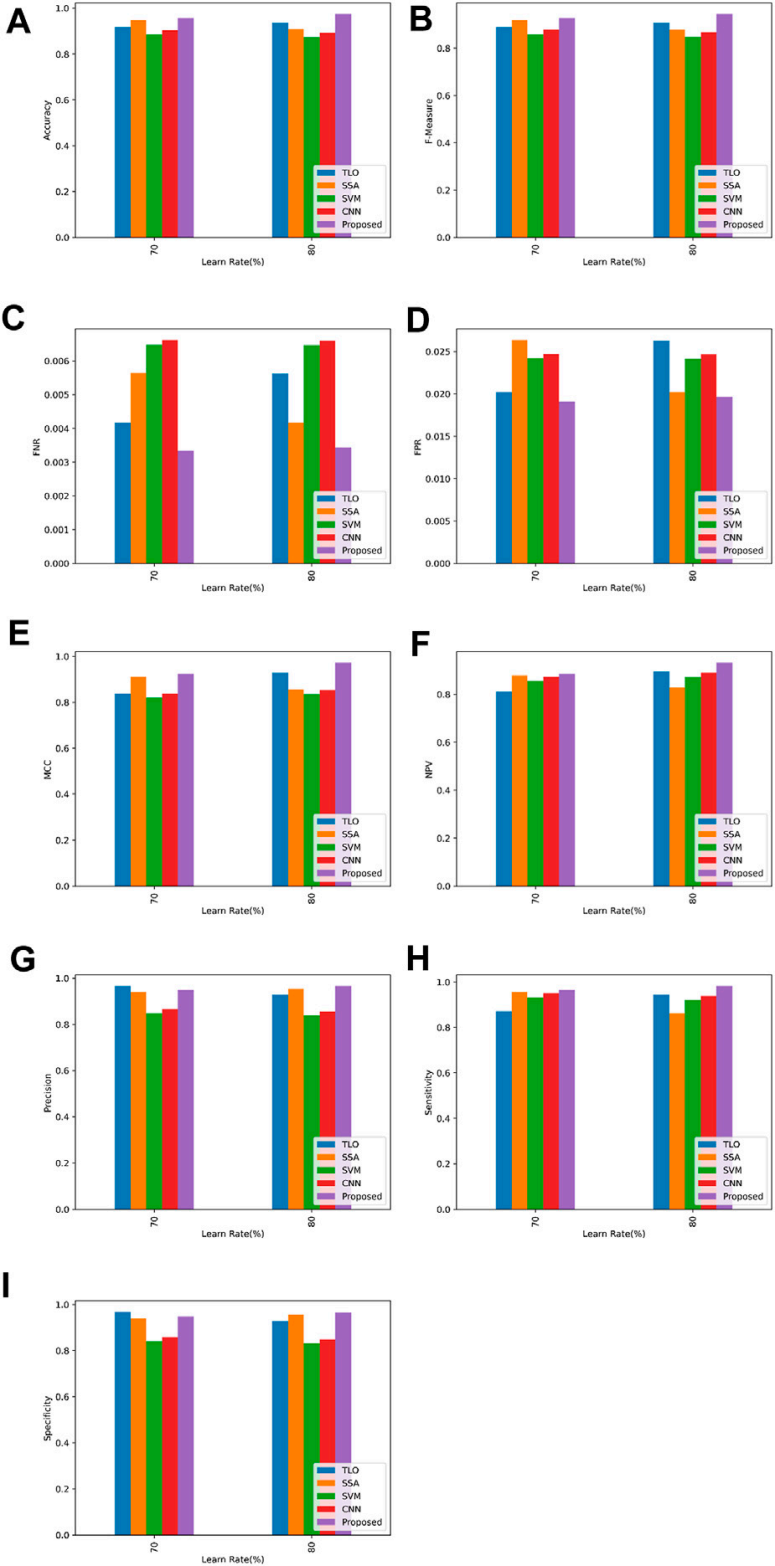
Overall Graphical Representation **(A)** accuracy **(B)** f-measure **(C)** FNR **(D)** FPR **(E)** MCC **(F)** NPV **(G)** precision **(H)** sensitivity **(I)** specificity.

Based on the findings, a graphical representation that shows how the proposed model and current articles compare in terms of classification performance is provided in Figure 5.

**FIGURE 6:**
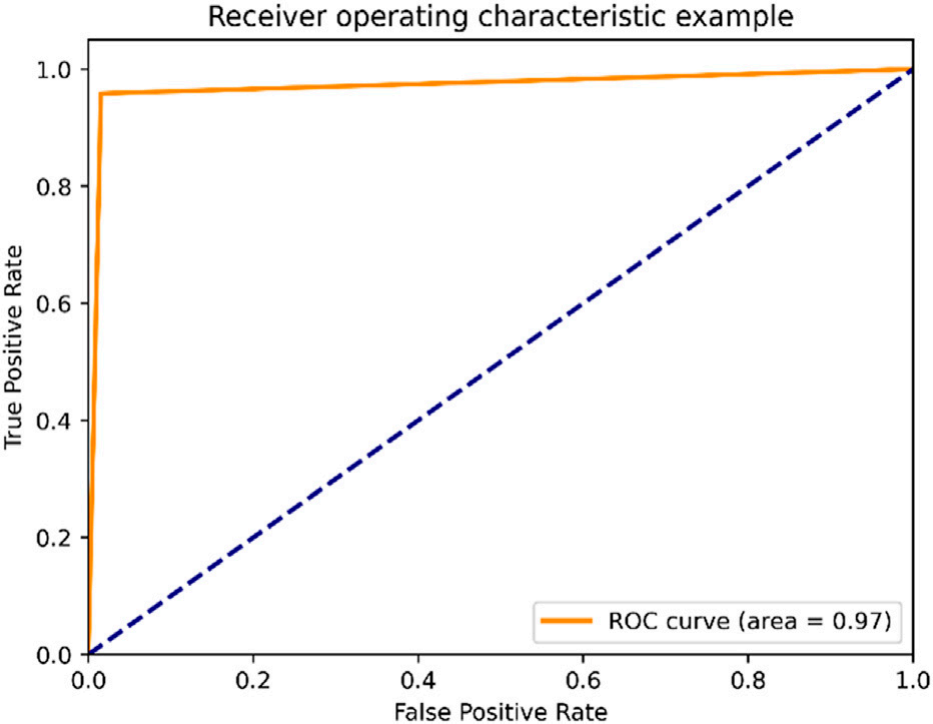
ROC curve.

Figure 6 shows the receiver operating characteristic curve, or ROC curve, is a graphic depiction that shows how well a classification model performs at various categorization criteria. The True Positive Rate (TPR) and False Positive Rate (FPR) are two important characteristics that are shown in connection to one another. The fraction of genuine positive events that the model properly classifies as positive is shown by the true positive rate, also known as sensitivity or recall. The ratio of true positives to the total of true positives and false negatives is used to compute it. On the other side, the False Positive Rate quantifies the percentage of real negative cases that the model misclassifies as positive. The ratio of false positives to the total is used to compute it. The trade-off between the True Positive Rate and the False Positive Rate by putting different threshold values on the ROC curve. The curve offers a visual depiction of the model’s performance throughout the whole range of potential thresholds, with each point on the curve denoting a different threshold. The performance of binary classification models may be assessed, and several models can be contrasted, using the ROC curve. It enables us to evaluate the model’s capacity for differentiating between favourable and unfavourable occurrences and to select an acceptable classification threshold based on the desired balance between sensitivity and specificity.

### 4.4 EDA analysis

**FIGURE 7:**
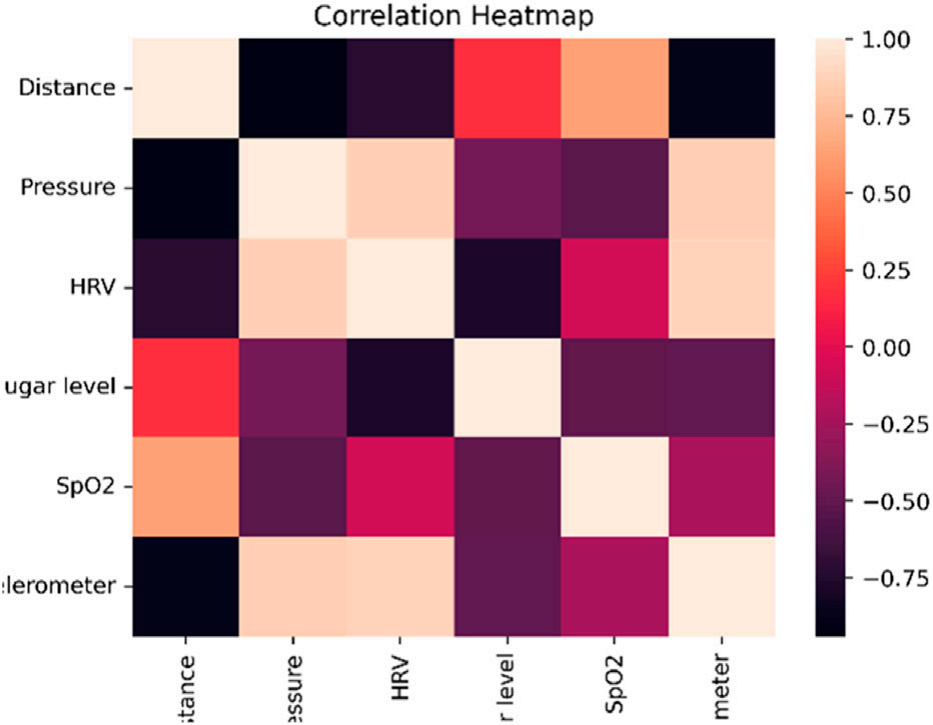
Correlation heatmap.

The links, patterns, and distributions within a dataset can be understood using EDA techniques including correlation matrix analysis, correlation heatmap visualisation, and pie chart depiction. They aid in understanding the data structure, locating relationships between variables, and understanding category proportions visually. Figure 7 shows the linear relationship between pairs of variables in a dataset is measured by the correlation matrix, which is shown graphically as a correlation heatmap. The correlation’s strength and direction are visually shown by color-coding. A quick and simple method of spotting associations between variables is the heatmap. Darker colours are often used to signify strong positive correlations, while lighter colours are typically used to represent strong negative correlations.

**FIGURE 8:**
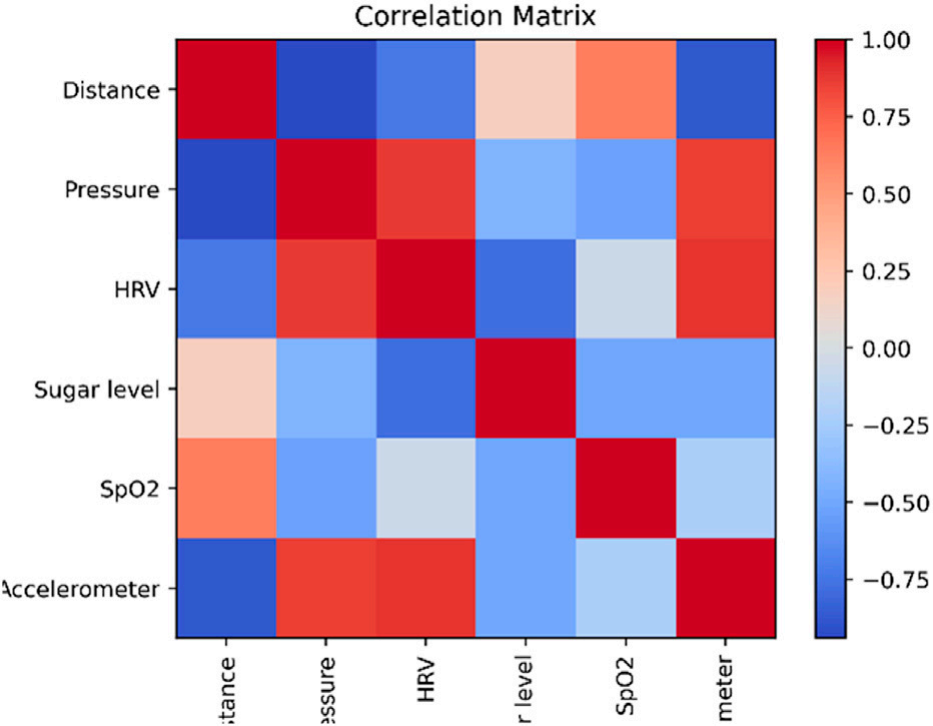
Correlation matrix.

The correlation coefficients between several variables in a dataset are displayed tabularly as a correlation matrix as shown in Figure 8. It offers a numerical analysis of the correlations between the different variables, highlighting their strength and direction. The correlation coefficients vary from −1 to 1, with a perfect negative correlation of −1, a perfect positive correlation of 1, and no connection at all at 0. Understanding the relationships and potential multicollinearity between variables is aided by a correlation matrix.

**FIGURE 9:**
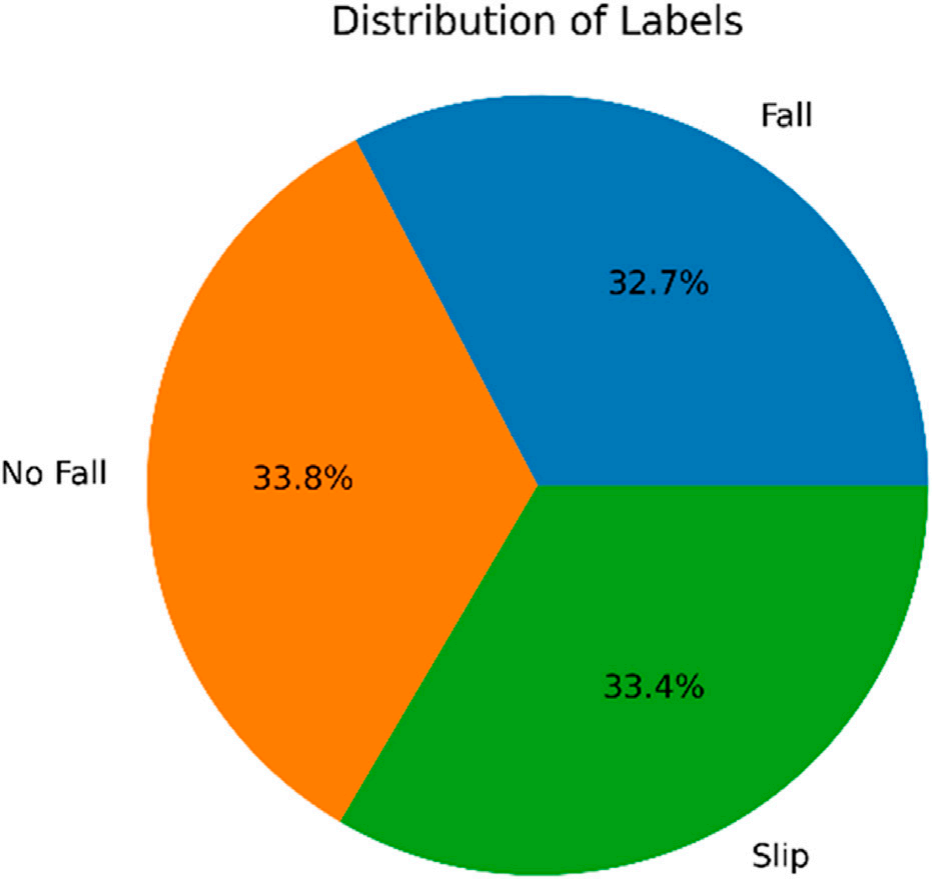
Pie-chart.

The proportion or percentage distribution of categorical variables in a dataset is shown by a circular statistical graphic with segments is called pie chart shown in Figure 9. The size of each pie slice, which represents a particular category in the data, is proportional to how frequently or how frequently relatively that category appears in the data. Pie charts are frequently used to show how categorical variables are composed or distributed and make it simple to compare categories.

Table 4 compares the performance metrics of a model trained with two different epoch sizes: 50 and 100. Epoch size refers to the number of times the learning algorithm iterates over the entire training dataset during training. The accuracy is 0.930878 for an epoch size of 50, and it rises to 0.955679 with an epoch size of 100. The precision increases from 0.930367 at an epoch size of 50 to 0.947812 at an epoch size of 100. With an epoch size of 50, the sensitivity is 0.945977, and with an epoch size of 100, it is 0.963715. The specificity is 0.930284 for an epoch size of 50 and rises to 0.947749 at an epoch size of 100. The F-measure is 0.908860 for an epoch size of 50 and 0.925902 for an epoch size of 100. A 50-epoch epoch has an MCC of 0.900408, and a 100-epoch epoch has an MCC of 0.923724. The NPV is 0.869601 for an epoch size of 50 and rises to 0.885927 at an epoch size of 100. The FPR is 0.026070 for an epoch size of 50, and it drops to 0.019067 with an epoch size of 100. At an epoch size of 50, the FNR is 0.005583, and at an epoch size of 100, it is 0.003342.

**TABLE 4:** Epoch size impact on performance: analysis.

Metrics	Epoch = 50	Epoch = 100
Accuracy	0.938078	0.955679
Precision	0.930367	0.947812
Sensitivity	0.945977	0.963715
Specificity	0.930284	0.947749
F-Measure	0.908860	0.925902
MCC	0.900408	0.923724
NPV	0.869601	0.885927
FPR	0.026070	0.019067
FNR	0.005583	0.003342

On several datasets referred to as Dataset1, Dataset2, and Dataset3, Table 5 offers a performance examination of various models, and Proposed model. For each model and dataset combination, the table gives measures like accuracy, precision, sensitivity, and specificity. With the best accuracy (0.974015), precision (0.965998), sensitivity (0.982206), and specificity (0.965933) among all models for Dataset1, the Proposed model exhibits outstanding performance. The Proposed model outperforms the TLO, SSA, SVM, and CNN models in every metric, while they all do rather well as well. The Proposed model continues to dominate with the greatest accuracy, precision, sensitivity, and specificity values across both datasets in Datasets 2 and 3. It outperforms other models, achieving accuracy scores of 0.958939 and 0.959167 for Datasets 2 and 3, respectively. All datasets show that the proposed model regularly outperforms competing models, proving its viability in reaching high performance metrics in sensor network settings.

**TABLE 5:** Performance analysis: varying dataset impact.

Metrics	TLO	SSA	SVM	CNN	Proposed
Dataset1 (Kaggle, 2023a)					
Accuracy	0.936500	0.908895	0.875339	0.893681	0.974015
Precision	0.928802	0.953798	0.838471	0.856041	0.965998
Sensitivity	0.944386	0.860979	0.920068	0.939347	0.982206
Specificity	0.928718	0.956176	0.831202	0.848619	0.965933
Dataset2 (Kaggle, 2023b)					
Accuracy	0.922004	0.894827	0.861790	0.879848	0.958939
Precision	0.914425	0.939035	0.825493	0.842791	0.951045
Sensitivity	0.929768	0.847652	0.905826	0.924807	0.967003
Specificity	0.914343	0.941376	0.818336	0.835484	0.950982
Dataset3 (The MobiFall and MobiAct datasets, 2023)					
Accuracy	0.922224	0.895040	0.861995	0.880058	0.959167
Precision	0.914643	0.939259	0.825690	0.842992	0.951272
Sensitivity	0.929990	0.847854	0.906042	0.925028	0.967233
Specificity	0.914561	0.941600	0.818531	0.835683	0.951208

## 5 Discussion

The proposed system aims to address the safety concerns of elderly individuals by developing a fall detection system empowered by IoT-Blockchain technology. The system utilizes sensor data from wearable devices, such as accelerometers and gyroscopes, to detect falls accurately. It combines LSTM, optimized CNN, and RNN models for fall detection and employs a hybrid optimization model (SSA + TLBO) to optimize the weights of the CNN. As shown in Table 1, there are currently few publicly accessible datasets that include a variety of human activities, including falls. This deficiency emphasises the need for fresh datasets that will enable impartial assessments of various fall detection techniques. The research community must also analyse and assess new machine learning methods. In order to meet these needs, propose the Elderly Fall Prediction and Detection dataset, a useful tool for carrying out experiments with a range of goals. Researchers can investigate and create novel fall detection strategies using this dataset. Wearable devices equipped with accelerometers and gyroscopes are worn by a diverse group of elderly participants. The data collection spans an extended period to capture various activities and potential falls comprehensively. Precise synchronization and timestamping are achieved through synchronized device clocks, ensuring accurate temporal alignment of sensor data streams. This meticulous approach enhances the reliability and accuracy of fall detection analysis. IoT sensors, including accelerometers and gyroscopes, are strategically worn by elderly individuals to capture movement data. Placed on wrists or belts, they communicate wirelessly via Bluetooth or Wi-Fi with a central system. This practical setup enables real-time monitoring and fall detection, enhancing the proposed solution’s viability for seamless integration into seniors’ living environments. Deploying the TriNet system faces challenges such as costs, sensor maintenance, user acceptance, privacy, and integration with healthcare systems. Ensuring user comfort and technical reliability, addressing cultural considerations, and providing support are crucial. Energy-efficient design and adaptability to real-world conditions are essential. Successful implementation requires careful budgeting, user education, and collaboration with healthcare providers to fully leverage the system’s benefits for elderly fall detection.

The selection of features, dataset properties, and dividing between training and testing data are some of the variables that affect how well machine and deep learning algorithms work. Due to differences in datasets, pre-processing procedures, classifiers, machine learning algorithms, acquisition devices, and testing locations, comparing studies becomes difficult. Two learning rates—70% and 80%—are used to evaluate the performance of the proposed system with that of the existing models. The suggested model performs better than the current models in both scenarios in terms of accuracy, achieving accuracy scores of 95.57% and 97.40% for the 70% and 80% learning rates, respectively. The proposed model achieves high precision scores of 94.78% and 96.59% for the 70% and 80% learning rates, respectively. The proposed model demonstrates high sensitivity scores of 96.37% and 98.22% for the 70% and 80% learning rates, respectively. The proposed model achieves specificity scores of 94.77% and 96.59% for the 70% and 80% learning rates, respectively. The proposed model achieves F-measure scores of 92.59% and 94.37% for the 70% and 80% learning rates, respectively. The proposed model achieves high MCC scores of 92.37% and 97.17% for the 70% and 80% learning rates, respectively. Implementing the practical fall detection system entails addressing tangible challenges. Battery life in wearables requires optimization to avoid user inconvenience. Balancing accurate fall detection with limited false alarms is crucial to prevent unnecessary distress. Evaluating costs against benefits is vital for economic viability, encompassing device expenses, network setup, and maintenance. User-friendly wearables that prioritize privacy compliance and data security ensure user acceptance. Seamless communication with emergency responders is essential for timely assistance. Real-world implementation hinges on effectively navigating these challenges, ensuring system usability, cost-effectiveness, and reliability, ultimately enhancing the safety and autonomy of elderly individuals.

While accuracy is indeed a pivotal criterion for evaluating the proposed model, a comprehensive assessment demands an array of metrics. Precision, measuring the proportion of correctly identified falls among predicted ones, and recall, capturing the fraction of actual falls correctly detected, provide insights into the system’s true positive performance. The F1-score harmonizes precision and recall, offering a holistic measure of model effectiveness. Sensitivity, specificity, and the Matthews Correlation Coefficient (MCC) illuminate the model’s balance between true positives and negatives. Furthermore, Negative Predictive Value (NPV), False Positive Rate (FPR), and False Negative Rate (FNR) shed light on error types and misclassifications. By embracing a multitude of metrics, our study comprehensively evaluates the proposed system’s performance across various scenarios and intricacies. This robust analysis strengthens the validity of our research, providing a nuanced understanding of how the fall detection model operates and excels under diverse conditions, ensuring its practical relevance and potential for widespread implementation.

The proposed fall detection system is evaluated using the “Elderly Fall Prediction and Detection” dataset, split into training and testing sets based on learning rates (70% and 80%). Data augmentation techniques like time shifting, amplitude scaling, and noise injection enhance dataset diversity. Performance metrics include accuracy, precision, recall, specificity, F-measure, NPV, FPR, FNR, and MCC, gauging system effectiveness. The system’s success relies on high accuracy, swift fall detection, low false positives/negatives. It is compared to existing methods, highlighting its superiority. The evaluation ensures the system’s ability to detect falls accurately, making elderly living safer.

In Table 6, the comparison of metrics between the base papers IFADS (Lu and Chu, 2018), FallViewer (Wang et al., 2021), cStick (Rachakonda et al., 2021), and the proposed approach is presented. The proposed method outperformed FallViewer and IFADS in terms of accuracy, obtaining a remarkable accuracy of 97.40%, surpassing the accuracies of IFADS, FallViewer, and cStick, respectively, of 95.96%, 95.8%, and 96.67%.

**TABLE 6:** Base paper comparison.

Metrics	IFADS (Lu and Chu, 2018)	FallViewer (Wang et al., 2021)	cStick (Rachakonda et al., 2021)	Proposed
Accuracy	95.96	95.8	96.67	97.40
Precision	93.94	-	-	96.59
Sensitivity	-	97	-	98.22
Specificity	-	93.4	-	96.59

### 5.1 Limitation

This article’s main drawback is the lack of testing the suggested method on actual fall incidents, which would validate its efficacy in identifying falls in practical situations. A major time and complexity cost are also added by extracting numerous cross-disciplinary time-series features. The suggested system’s lack of performance testing in actual fall circumstances, which restricts its ability to be applied practically and its ability to be considered reliable, is an important drawback.

### 5.2 Future scope

Future research must take into account the high time and complexity costs involved in extracting a variety of cross-disciplinary time-series features. It would be beneficial to look into ways to simplify the feature extraction process and cut down on computational overhead. Researchers can reduce the complexity and time demands while preserving or enhancing the fall detection system’s effectiveness by optimising feature selection and extraction methods.

## 6 Conclusion

Falling occurrences posed a serious difficulty for the many older persons who lived alone in their houses since they frequently found it difficult to ask for aid. Because of the increase in the number of senior citizens, fall accidents have become a critical public health concern. This study’s goal was to create a blockchain-based IoT fall detection system for the elderly. Six key stages made up the suggested model: data collection, preprocessing, feature extraction, feature selection, fall detection, and emergency response and assistance. Elderly wearable devices with accelerometers and gyroscopes had their sensor data gathered. Preprocessing procedures were used to handle missing and null values for the acquired data. Autocorrelation, Statistical characteristics, and Principal Component Analysis (PCA) were used to extract features following preprocessing. SSA and TLBO, a novel hybrid technique, were used to choose the best features. The goal of this strategy, known as HSSTL, was to enhance feature selection. The proposed model also included TriNet, a network made up of LSTM, an enhanced CNN, and RNN, for reliable fall detection. The optimised CNN generated using the HSSTL hybrid optimisation model was employed to improve fall detection accuracy. Additionally, when a fall happened, the data for fall detection was safely kept in the Blockchain network. Blockchain network was used to warn nearby residents, family members, or other important parties who needed immediate assistance. The system’s functionality and the required outcomes were realised by implementing the proposed model using the PYTHON programming language. At a 70% learning rate, the suggested model excelled with an accuracy score of 0.955679, while the proposed model outperformed at an 80% learning rate with an accuracy of 0.974015. In conclusion, this study introduces a novel fall detection system for the elderly, harnessing IoT, advanced machine learning, and blockchain technologies. Key contributions include a hybrid feature selection approach, TriNet architecture, and secure data storage. Future work could focus on refining model robustness, extending to larger datasets, and integrating more IoT devices to further enhance fall detection accuracy and real-world applicability.

## Data Availability

The original contributions presented in the study are included in the article/Supplementary Material, further inquiries can be directed to the corresponding author.
