# Supplementary figures and images for: IoT-blockchain empowered Trinet: optimized fall detection system for elderly safety
Source: Front Bioeng Biotechnol. 2023 Sep 21;11:1257676. doi: 10.3389/fbioe.2023.1257676 (PMC10552752; doi:10.3389/fbioe.2023.1257676)

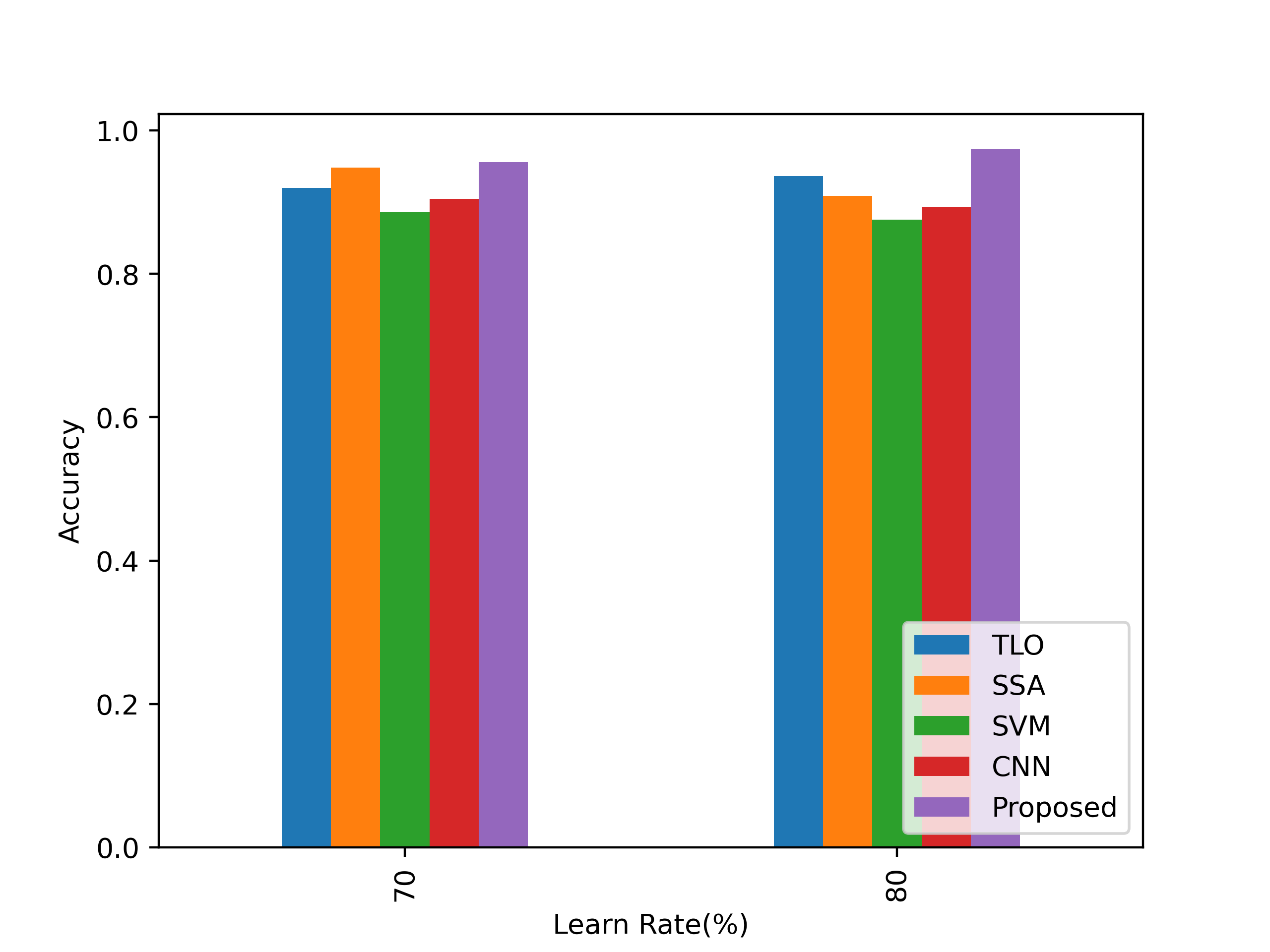

Supplement: Supplementary file 1 [file DataSheet1.ZIP › sourcecode/Results/Accuracy.png]

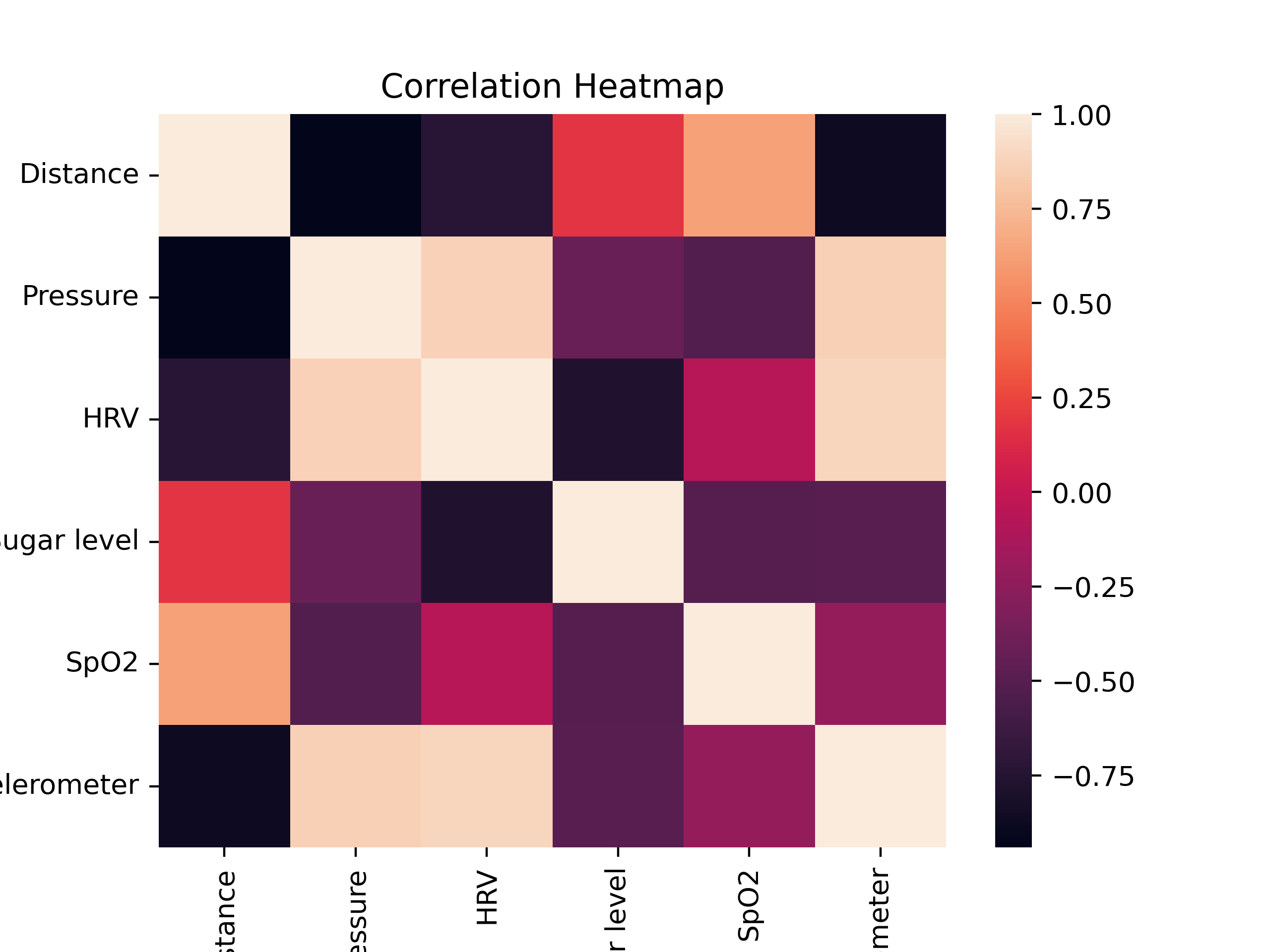

Supplement: Supplementary file 1 [file DataSheet1.ZIP › sourcecode/Results/Correlation Heatmap.png]

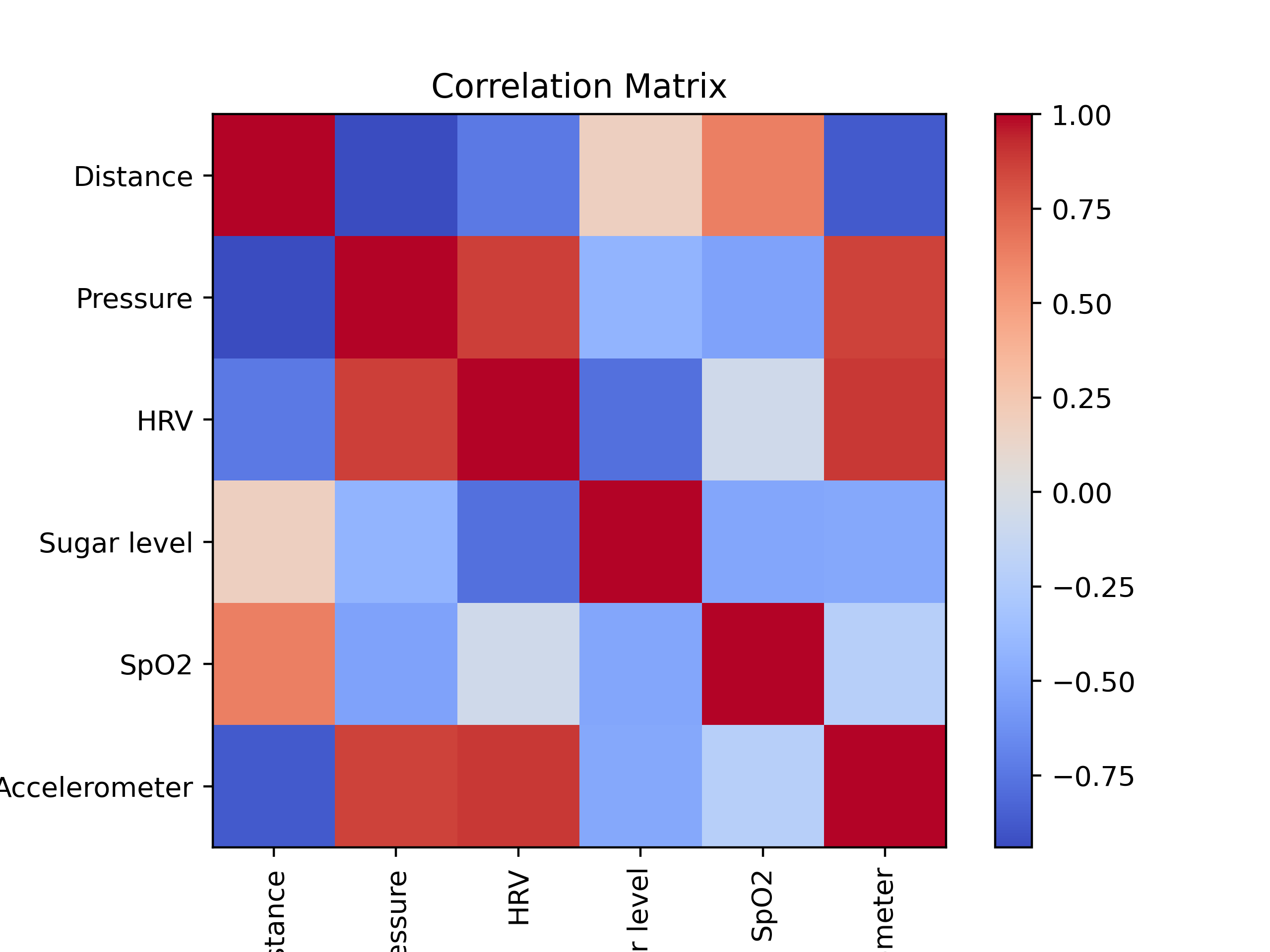

Supplement: Supplementary file 1 [file DataSheet1.ZIP › sourcecode/Results/Correlation Matrix.png]

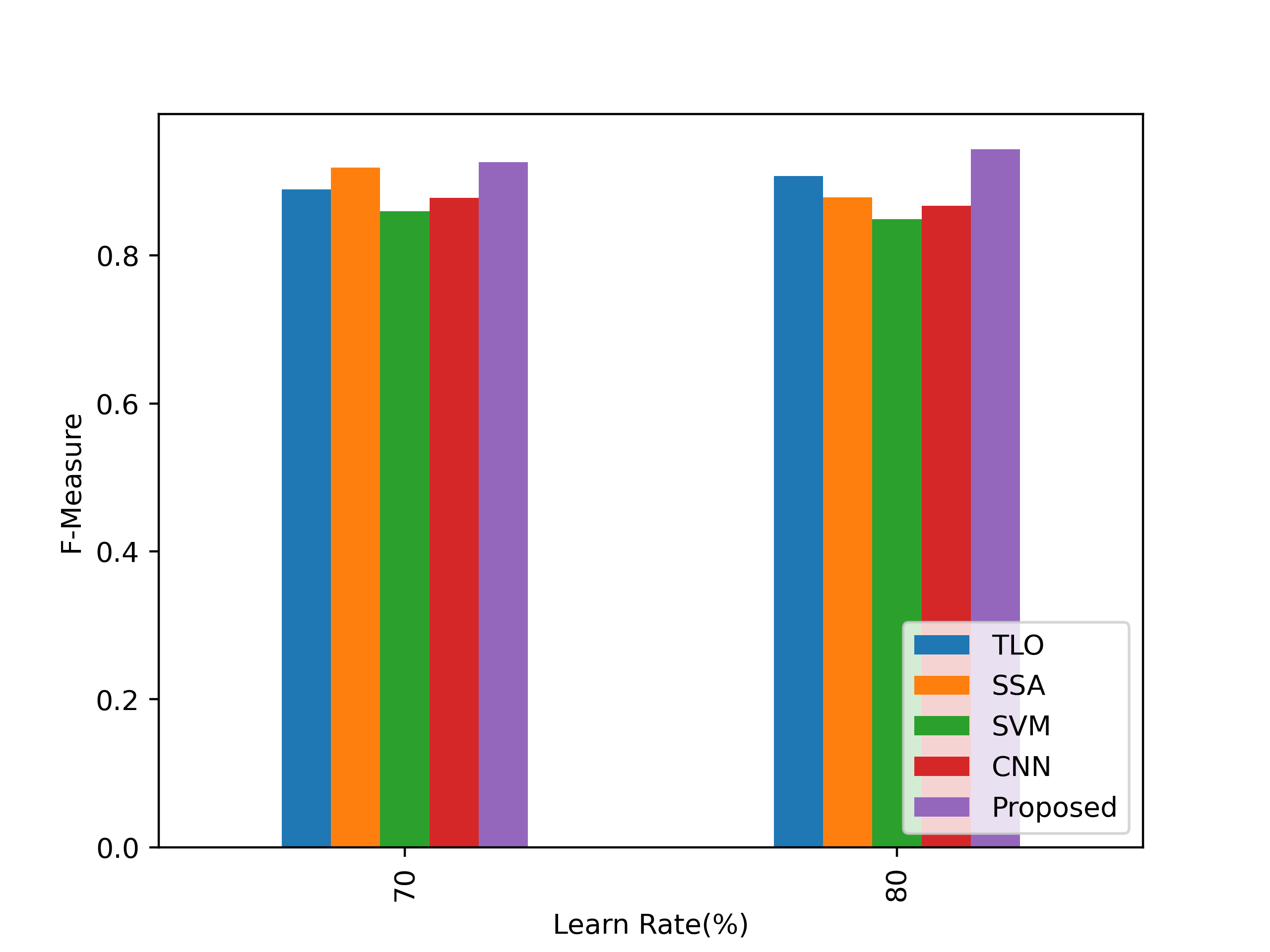

Supplement: Supplementary file 1 [file DataSheet1.ZIP › sourcecode/Results/F-Measure.png]

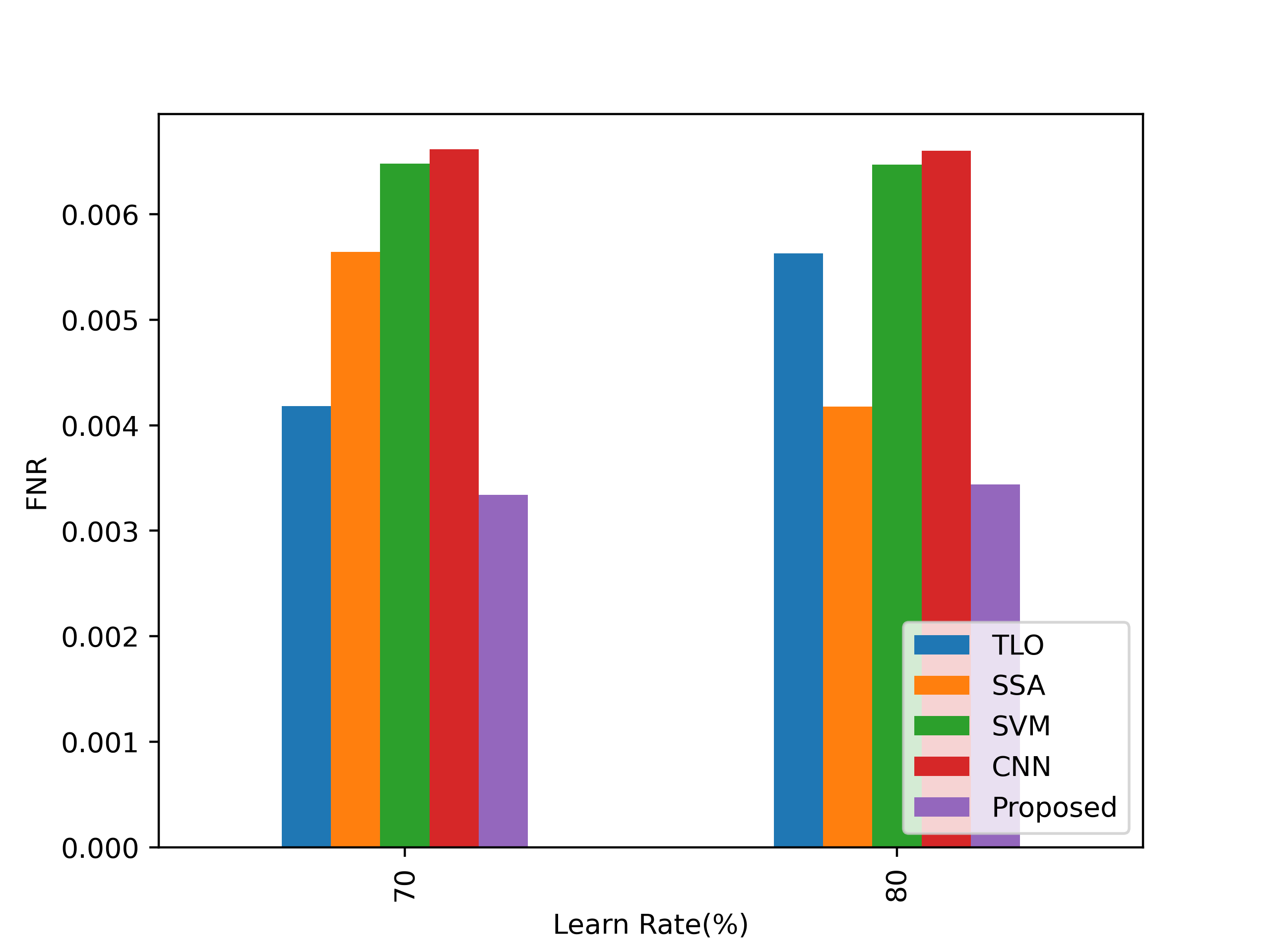

Supplement: Supplementary file 1 [file DataSheet1.ZIP › sourcecode/Results/FNR.png]

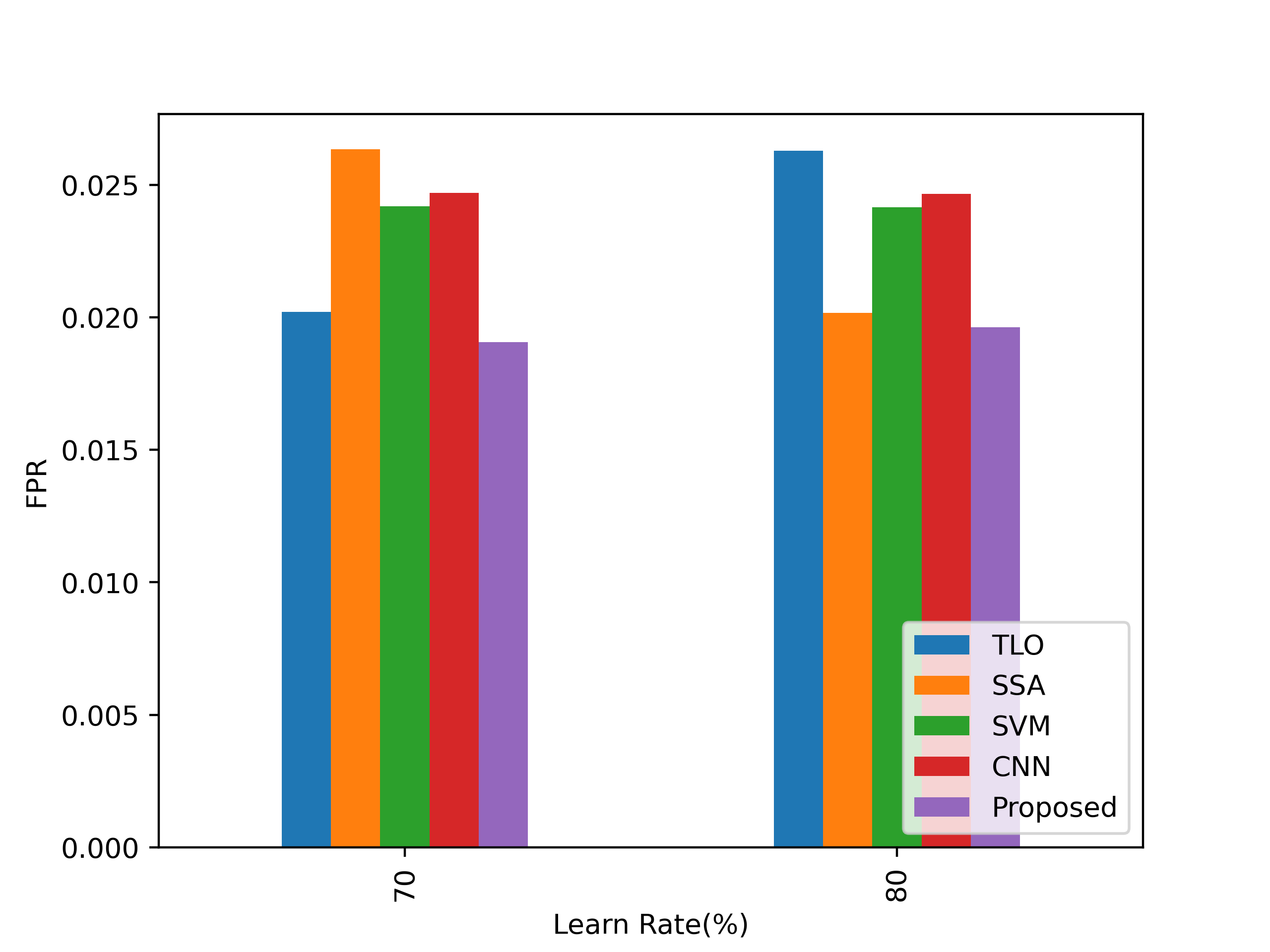

Supplement: Supplementary file 1 [file DataSheet1.ZIP › sourcecode/Results/FPR.png]

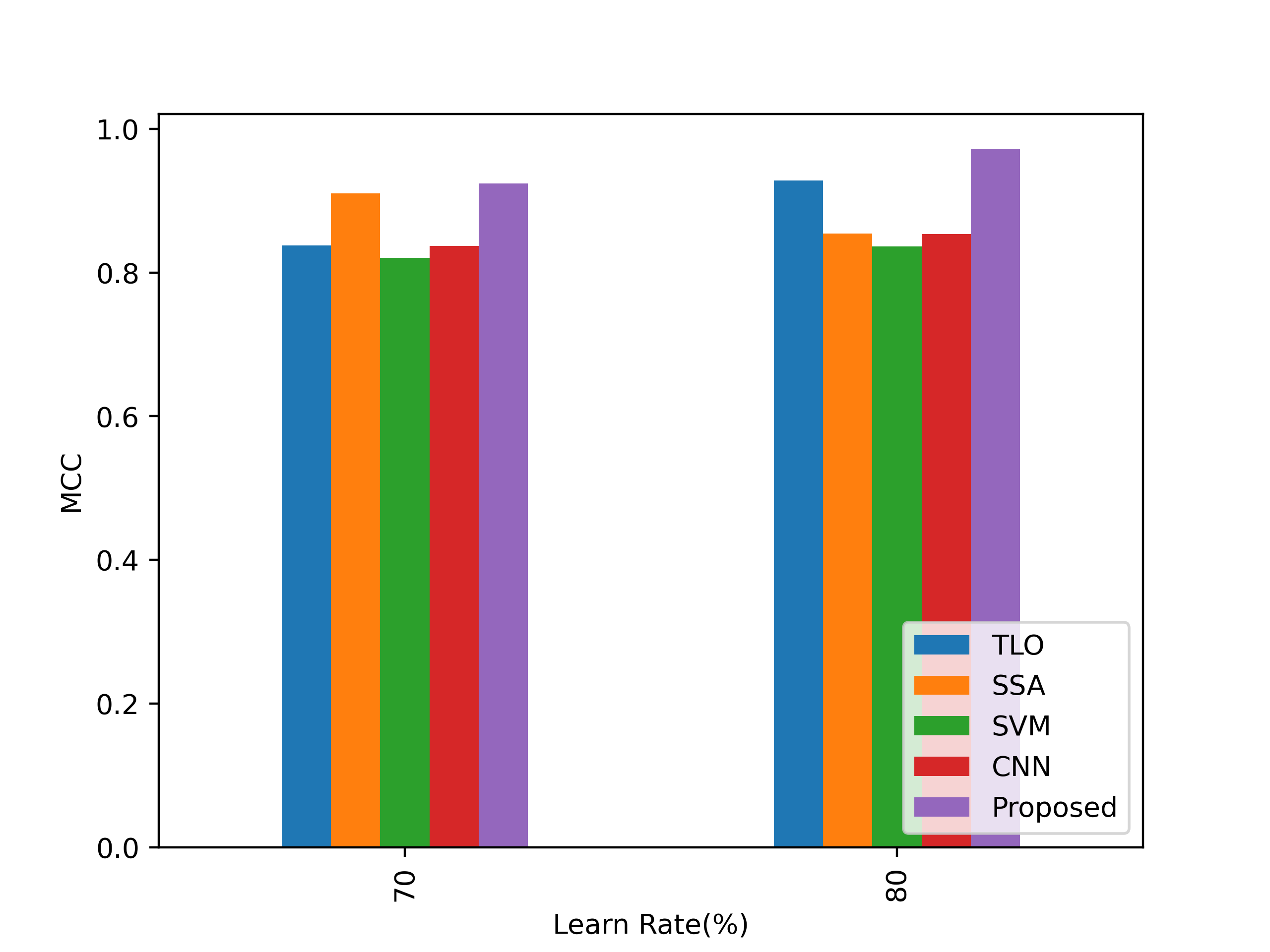

Supplement: Supplementary file 1 [file DataSheet1.ZIP › sourcecode/Results/MCC.png]

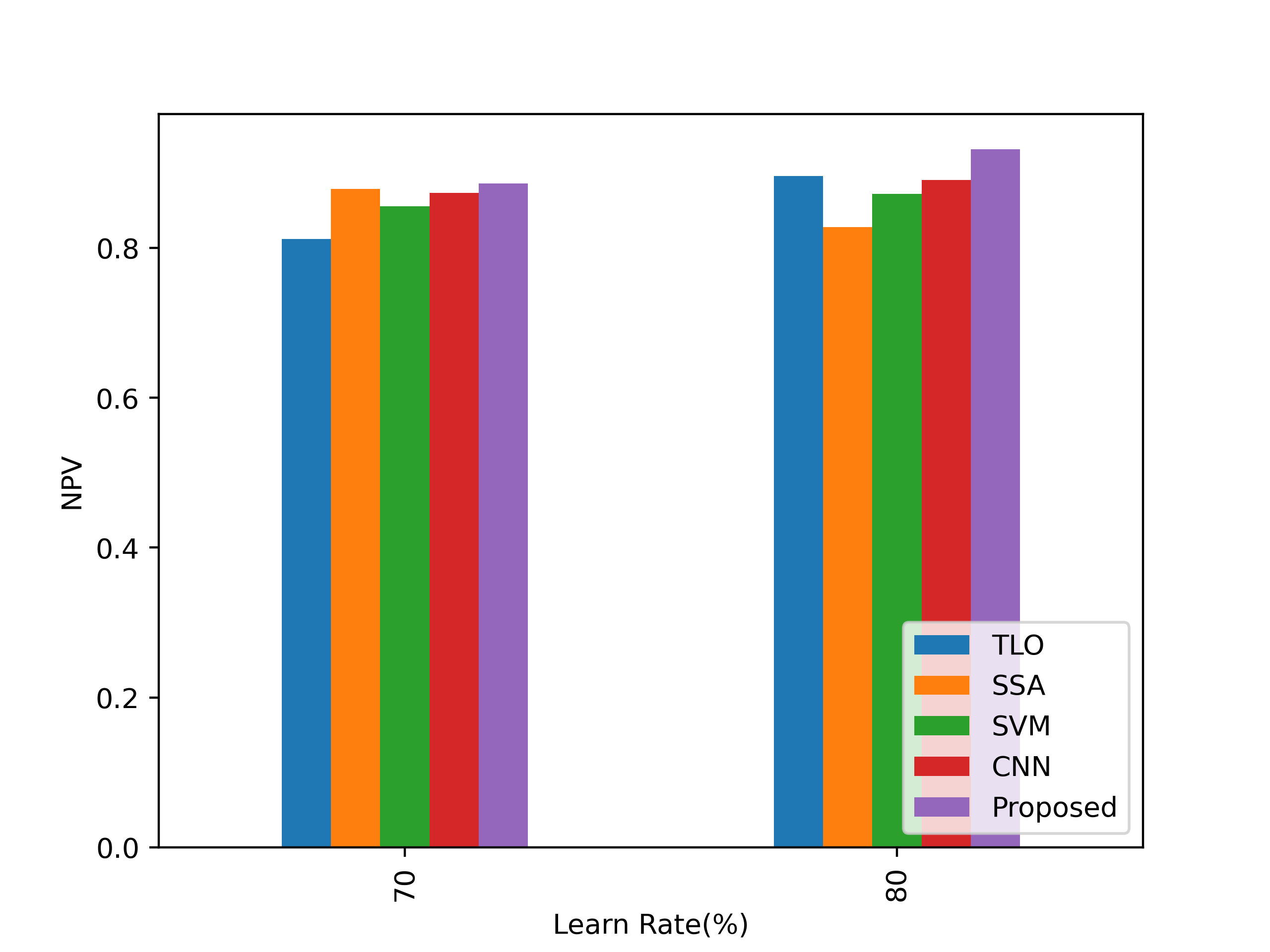

Supplement: Supplementary file 1 [file DataSheet1.ZIP › sourcecode/Results/NPV.png]

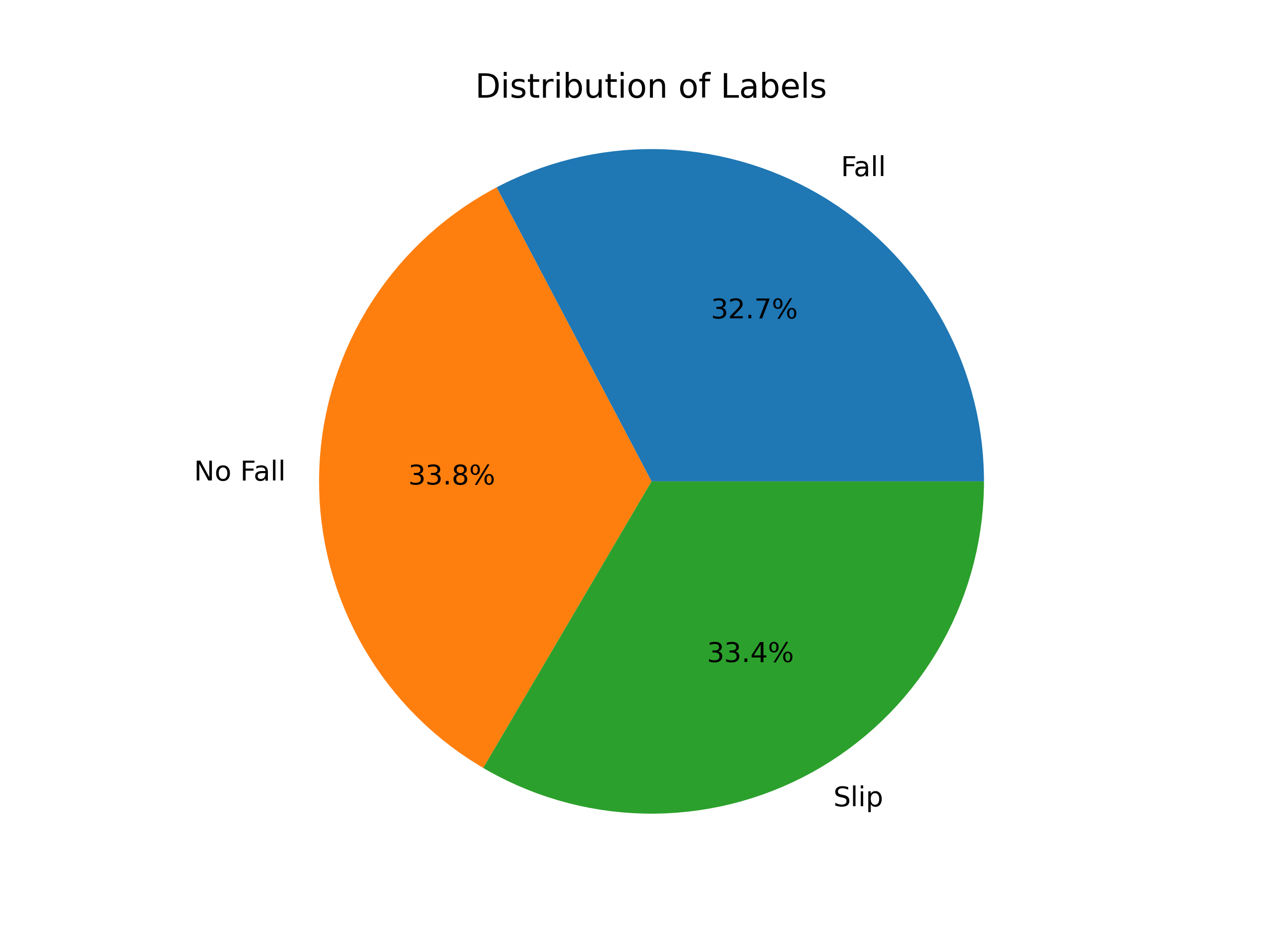

Supplement: Supplementary file 1 [file DataSheet1.ZIP › sourcecode/Results/piechart.png]

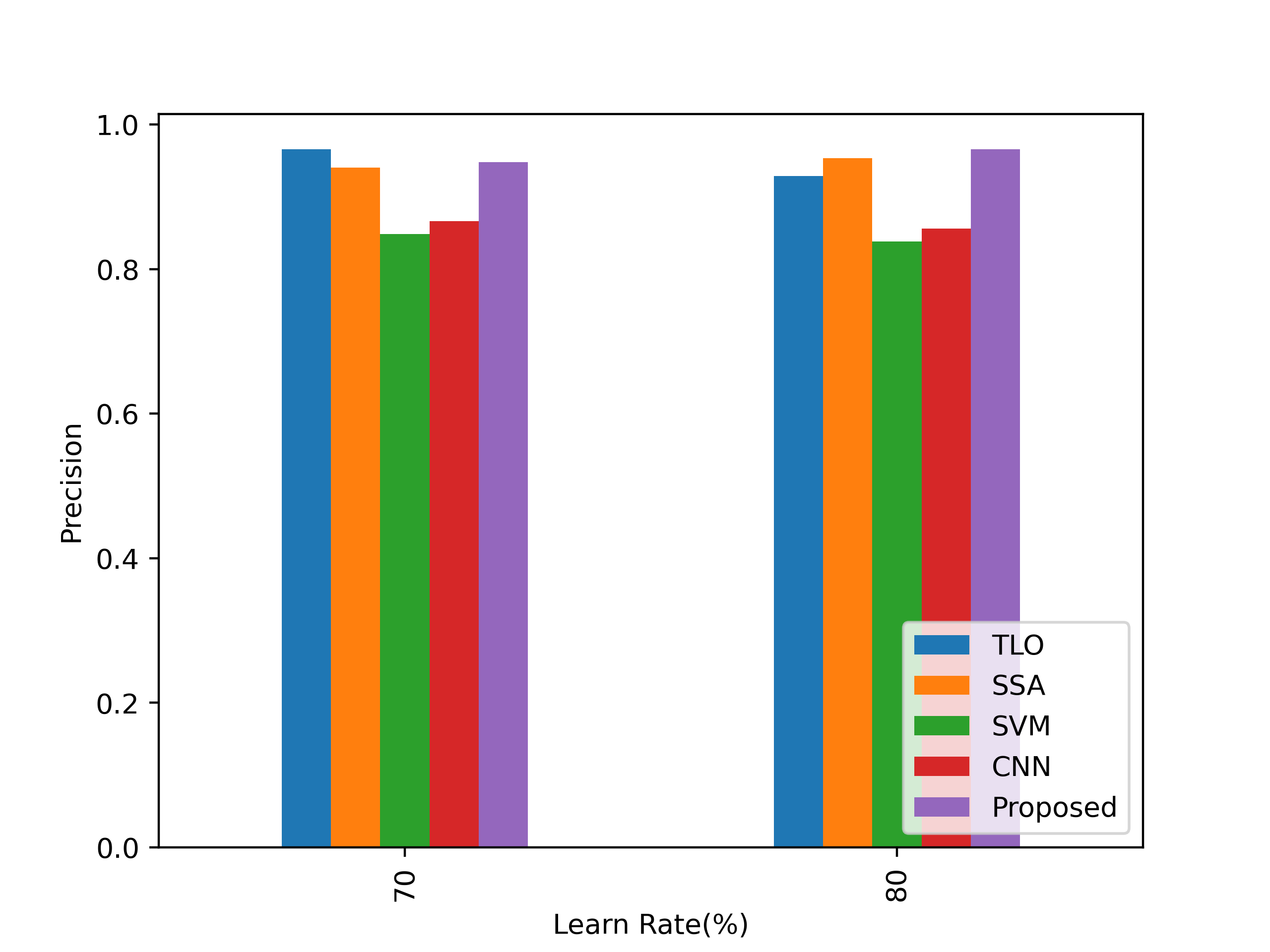

Supplement: Supplementary file 1 [file DataSheet1.ZIP › sourcecode/Results/Precision.png]

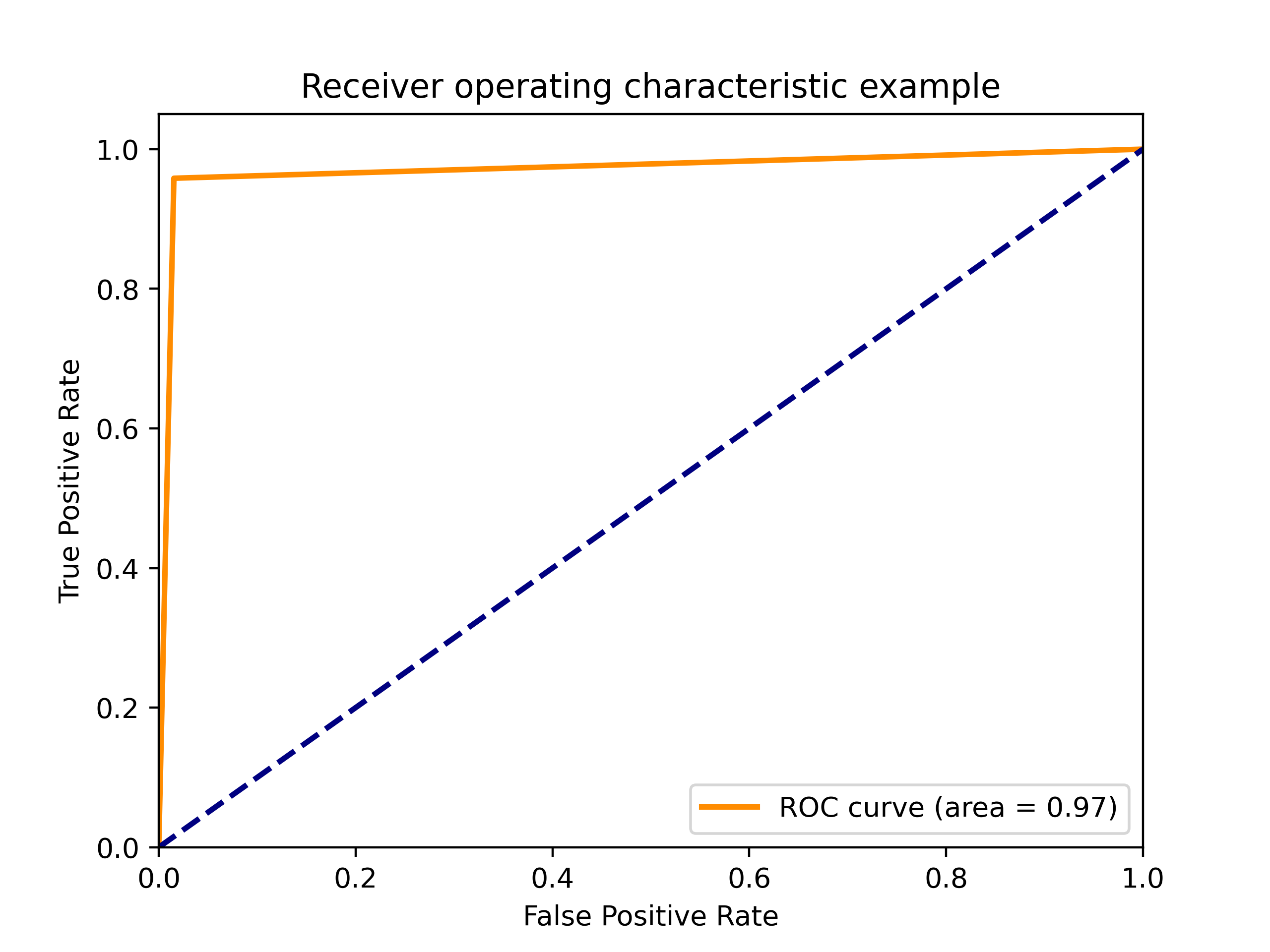

Supplement: Supplementary file 1 [file DataSheet1.ZIP › sourcecode/Results/roc_curve.png]

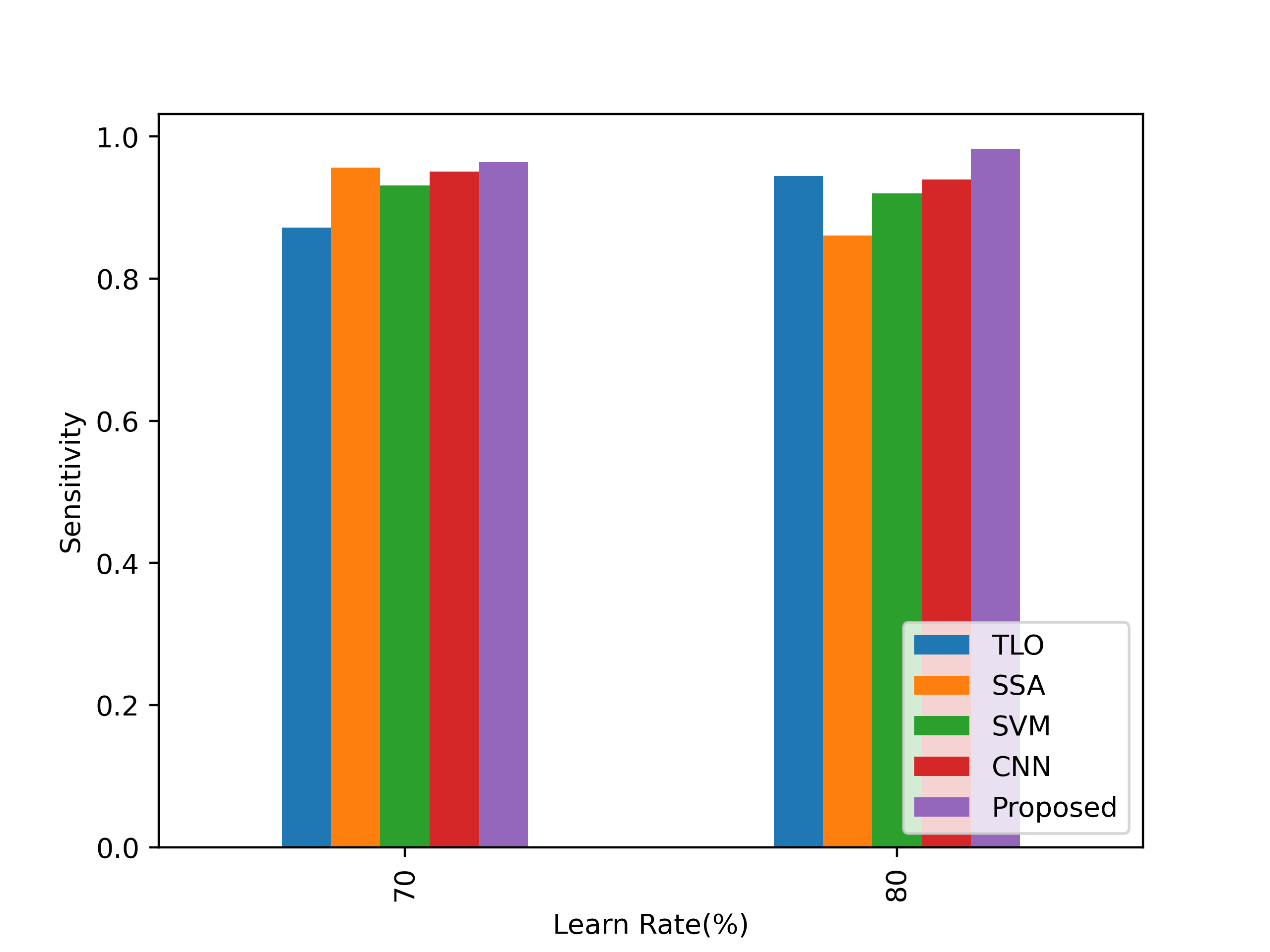

Supplement: Supplementary file 1 [file DataSheet1.ZIP › sourcecode/Results/Sensitivity.png]

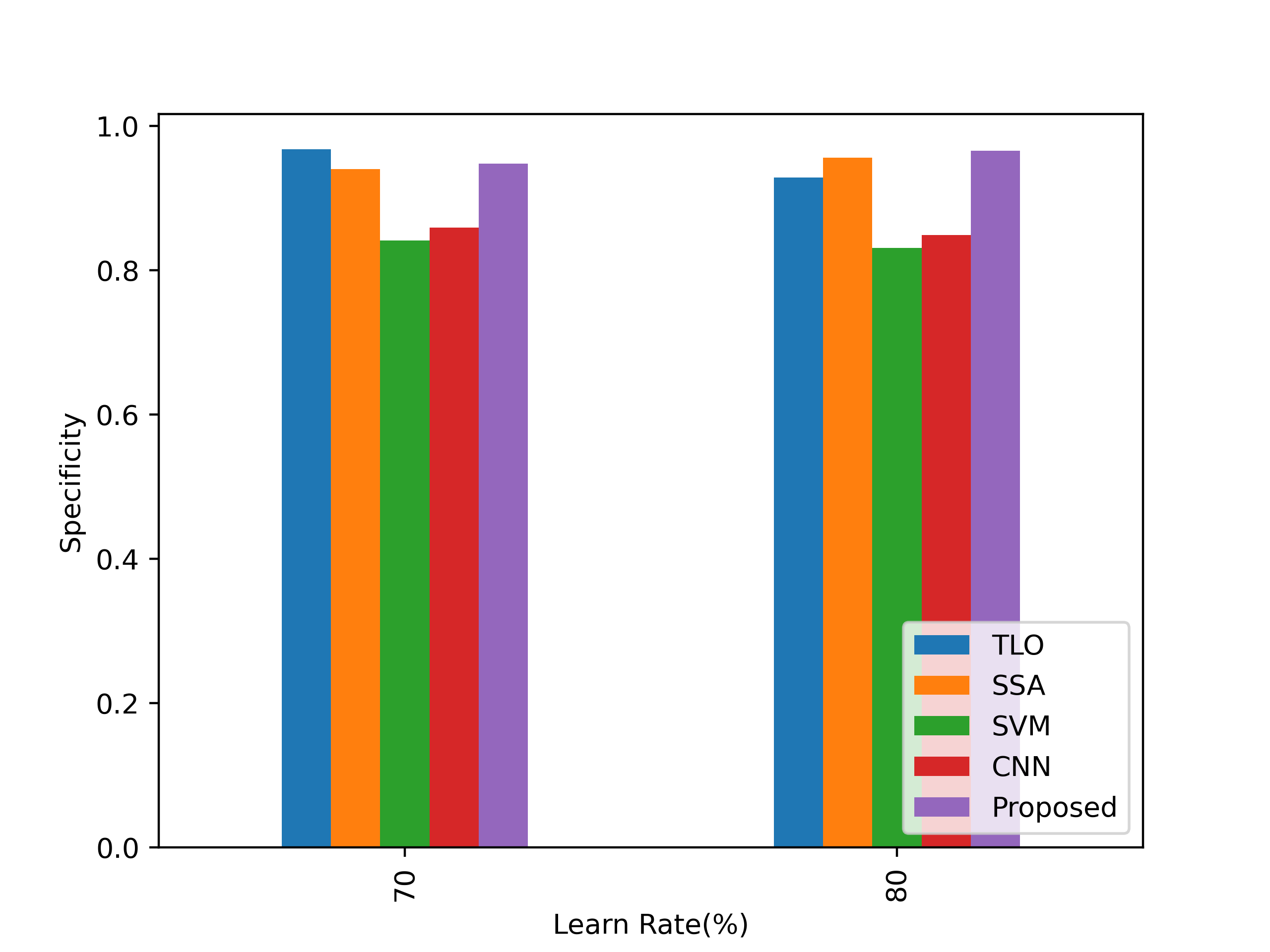

Supplement: Supplementary file 1 [file DataSheet1.ZIP › sourcecode/Results/Specificity.png]
